# Bacterial-Based Cancer Therapy (BBCT): Recent Advances, Current Challenges, and Future Prospects for Cancer Immunotherapy

**DOI:** 10.3390/vaccines9121497

**Published:** 2021-12-18

**Authors:** Kajal H. Gupta, Christina Nowicki, Eileena F. Giurini, Amanda L. Marzo, Andrew Zloza

**Affiliations:** 1 Division of Hematology, Oncology, and Cell Therapy, Department of Internal Medicine, Rush University Medical Center, Chicago, IL 60612, USA; Kajal_Gupta@rush.edu (K.H.G.); Christina_A_Nowicki@rush.edu (C.N.); Eileena_F_Giurini@rush.edu (E.F.G.); Amanda_Marzo@rush.edu (A.L.M.); 2 Division of Translational and Precision Medicine, Department of Internal Medicine, Rush University Medical Center, Chicago, IL 60612, USA

**Keywords:** bacterial therapy, bacteriotherapy, tumor targeting bacteria, cancer immunotherapy, therapeutic bacteria

## Abstract

Currently approximately 10 million people die each year due to cancer, and cancer is the cause of every sixth death worldwide. Tremendous efforts and progress have been made towards finding a cure for cancer. However, numerous challenges have been faced due to adverse effects of chemotherapy, radiotherapy, and alternative cancer therapies, including toxicity to non-cancerous cells, the inability of drugs to reach deep tumor tissue, and the persistent problem of increasing drug resistance in tumor cells. These challenges have increased the demand for the development of alternative approaches with greater selectivity and effectiveness against tumor cells. Cancer immunotherapy has made significant advancements towards eliminating cancer. Our understanding of cancer-directed immune responses and the mechanisms through which immune cells invade tumors have extensively helped us in the development of new therapies. Among immunotherapies, the application of bacteria and bacterial-based products has promising potential to be used as treatments that combat cancer. Bacterial targeting of tumors has been developed as a unique therapeutic option that meets the ongoing challenges of cancer treatment. In comparison with other cancer therapeutics, bacterial-based therapies have capabilities for suppressing cancer. Bacteria are known to accumulate and proliferate in the tumor microenvironment and initiate antitumor immune responses. We are currently well-informed regarding various methods by which bacteria can be manipulated by simple genetic engineering or synthetic bioengineering to induce the production of anti-cancer drugs. Further, bacterial-based cancer therapy (BBCT) can be either used as a monotherapy or in combination with other anticancer therapies for better clinical outcomes. Here, we review recent advances, current challenges, and prospects of bacteria and bacterial products in the development of BBCTs.

## 1. Introduction

Almost a century since Dr. William Coley made his first attempt to use bacterial products as immunotherapy, the use of live, attenuated bacteria has become a promising alternative to combat cancer [1,2]. Ongoing progress in understanding various molecular and cellular immunology of bacterial physiology associated with host–pathogen interactions has helped in the design of attenuated bacteria as conventional vaccine vectors [3]. In recent years, with developments in technology and our ability to attenuate pathogenic strains, research has been mainly focused on biochemical and molecular techniques by which bacteria can be manipulated in the fight against cancer. Bacteria have been of particular interest due to their natural motile ability, which allows them to move away from the vasculature and penetrate hypoxic regions of the tumor [4] and subsequently proliferate within tumor cells [5]. This solves the problem commonly faced by chemotherapeutics where they reach mainly the vascularized outside edges of the tumor but not the hypoxic core. Additionally, bacteria can be genetically modified to carry and express therapeutic proteins and tumor-associated antigens (TAAs), deliver genes, or transport chemotherapeutic molecules [6]. Direct delivery of drugs via bacteria to the tumor site enhances specific cancer-targeting therapies and limits the negative effects of treatment [7]. Alternatively, bacteria can also be harnessed to produce drugs within tumor cells, essentially manufacturing therapeutic molecules on site [8]. While our insights into cancer-specific treatments have vastly increased, there is still room for finding better targets for cancer therapies, possibly through the manipulation of known microorganisms [9]. Various bacterial species have proven useful in harnessing antitumoral immunity by initiating innate and adaptive immune responses in pre-clinical and clinical studies, which has increased the chances of tumor elimination without additional secondary side effects [10,11,12]. Microorganisms host a variety of mechanisms with potential in cancer therapy, many of which we have yet to be discovered and studied in detail. Recently, many bacterial therapeutics have been implemented in human clinical trials (phase I/II) [13,14,15,16,17,18]. In this article, we review recent advances in bacterial-mediated drug and delivery systems’ discovery and discuss the benefits and current challenges in these serving as anti-cancer treatments. We also discuss how various pathogenic and non-pathogenic bacteria have been genetically manipulated to induce tumor regression and the prospects of BBCT.

## 2. Bacterial Components and Products Targeted via BBCT

**Figure 1:**
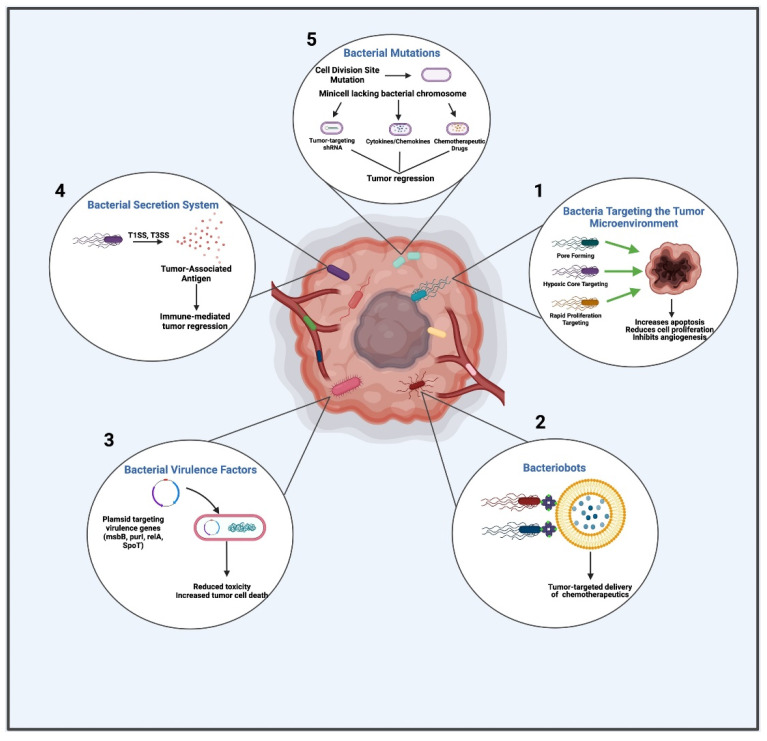
Schematic summary of the various bacterial mechanisms utilized in BBCT. (1) Anaerobic facultative bacteria specifically target the hypoxic environment of tumors initiating an inflammatory reaction resulting in tumor destruction. (2) Bacteriobots for cancer therapy, which involve targeting controlled drug release, improved cell adhesion, and improved penetration into the cell. (3) Bacterial virulence factors (e.g., msbB, purl, relA, SpoT) can be bioengineered to reduce toxicity and increase tumor cell death. (4) Bacterial toxins, such as the bacterial secretory system (T1SS and T3SS), can be used to inhibit the growth of solid tumors. (5) Bacterial mutations help delivery of immunomodulators such as cytokines, chemokines, and small molecules along with immune checkpoint antibodies, which can stimulate anti-tumor responses. This figure was created using Biorender.com.

Advancement of BBCT lies in focusing on cancer cells by different mechanisms that target a specific bacterial component or machinery. These mechanisms responsible for anti-cancer activity include targeting the tumor microenvironment, secretion of cytotoxic agents, manipulating bacterial virulence agents, and engineered bacterial vectors for the expression and release of tumoricidal proteins. Figure 1 depicts the overall mechanistic overview of BBCT.

### 2.1. Bacterial Targeting of the Tumor Microenvironment

One of the main driving factors for the use of bacterial-targeted drug delivery is related to the ability of anaerobic species to thrive in hypoxic tumor cores [19]. The tumor microenvironment is characterized by oxygen concentrations ≤ 10 mmHg [20]. Additionally, acidity primed by lactic acid results as a byproduct of metabolism of anaerobic bacteria because of decreased oxygen [21]. Further, the tumor microenvironment has increased tissue necrosis, resulting from tumor cell death due to the lack of nutrients and uncontrolled growth [4]. Hypoxia is a trademark of quickly proliferating solid tumors, a characteristic attributed to their expanding beyond the available blood supply [22]. The structure of blood vessel vasculature is functionally abnormal in tumors, resulting in irregular blood flow throughout the tissue, leading to oxygen deprivation [23]. The hypoxic condition forces tumors to develop adaptive genetic changes that withstand hypoxia-induced cell death and tissue necrosis [24]. The hypoxic tumor region is known to be associated with a higher expression of MDR1 (a multidrug-resistant gene) and P-glycoprotein genes, which are responsible for the development of multidrug resistance to various anticancer drugs [25].

However, the hypoxia caused by these poorly organized blood vessels creates a unique niche for anaerobic bacteria to flourish [9]. Therefore, areas of tumors that before were most resistant to chemotherapy can now be specifically targeted through the use of microorganisms as drug and gene delivery systems [26]. It has been shown that growth and survival of bacteria in tumors is dependent on their mechanisms for motility and survival, as well as their level of dependence on oxygen [27,28]. It has been previously demonstrated that *Salmonella* sp. and *Clostridia* sp. preferentially target and replicate in the core anaerobic zone of tumors [29] (the specific targets of these bacteria are discussed further in this review). Thus, bacteria pose a possible solution to the issue of specificity in drug and gene delivery of cancer therapy.

### 2.2. Bacteriobots

“Bacteriobots” are devices designed to use bacteria as microactuators and micro-sensors to deliver various types of chemotherapeutics and other therapeutic compounds to the inner and invasive layers of the tumor [30,31]. Bacteriobots are designed to regulate the speed and migration to direct the chemotaxis of bacteria towards the tumor site. The targeted tumor is attacked by bacteriobots that adhere to the cancer cells and are engineered to secrete anti-tumor agents, further destroying the tumor [32]. Park et al. demonstrated motility of bacteriobots constructed by measuring the binding of biotin displayed on the outer membrane proteins of the bacterium *S. Typhimurium* and streptavidin, which was coated on the surface of drug-loaded liposomes [33]. Various other bacteria such as *S. marcescens E. coli*, magnetotactic bacteria, and *S. Typhimurium* have been employed to develop bacteria-based microbots. Their clinical applications, however, are limited due to high pathogenicity and acquired antibiotic resistance, as well as difficult expansion and specific nutritional requirements. However, soon we may expect to have bacteriobots designed with a tumor-targeting bacteria, for use as a biomedical and clinical microrobot for cancer diagnosis and therapy.

### 2.3. Bacterial Virulence Factors

Virulence factors are cellular structures, molecules, and regulatory systems that enable microbial pathogens to achieve colonization and growth within the host, immune evasion, and immunosuppression, as well as entry and exit out of cells and extraction of nutrition from cancer cells [34,35]. Thus, it is highly essential to normalize the bacterial virulence against the host immune system. However, some virulence factors can be responsible for the anti-tumor response, and, thus, deleting or manipulating these virulence factors can reduce anti-cancer effects of the bacteria. Thus, it is important to attenuate a strain without altering the anti-tumor activity. *Salmonella Typhimurium* strain VNP20009, which has been broadly studied for its anti-tumor specificity, is altered by deleting major virulence genes, including *msbB* and *purI* [36]. Deletion in *msbB* gene leads to myristoylation of the lipid A component of LPS, which induces TNF production and can reduce the risk of sepsis. Mutations in other genes like *rfaG* and *rfaD* result in the production of truncated LPS in the host, which in turn leads to the reduction of toxicity and generates a productive anti-tumor response [37]. Mutants made by deleting relA- and SpoT from *Salmonella* spp. are impaired in the synthesis of ppGpp, a signaling molecule known to be involved in gene expression stringent response in bacteria; however, the mutant strain exhibits less toxicity. The ΔppGpp strain is known to have anti-tumor responses and to activate inflammasome NLRP3 and IPAF as well as the expression of many pro-inflammatory cytokines [38]. 

*Listeria monocytogenes’* cytotoxicity can be altered by deleting genes that are involved in cell invasion. *Hyl* gene deletion can cause defects in phagolysosome release [39,40]; and mutation in gene *actA* or *ActA* PEST-like sequences abrogates intracellular diffusion [41,42], and mutant strains *inlA* and *inlB* lack properties of invasion [43,44]. *Clostridium* spp. infection induces a variety of secreted toxins, such as actin-specific ADP-ribosyltransferase, hemolysins, phospholipases, and other pore-forming toxins, which interfere with intracellular functions [45,46]. 

### 2.4. The Bacterial Secretion System 

Bacteria employ secretion systems to transport virulence proteins, which can be manipulated and exploited for novel cancer treatments. Essentially, this involves signaling molecules that are necessary for delivery in a bacterial secretion system and then fusing therapeutic molecules to them for more efficient and targeted drug delivery [47]. A secretion system that is commonly taken advantage of in cancer therapy is the type III secretion system (T3SS), which acts by directly injecting bacterial proteins into the host cell cytoplasm [48]. The efficacy of T3SS for drug delivery has been the focus of several studies, genetically fusing T3SS with tumor-associated antigen, Survivin, resulting in complete tumor regression [49,50]. The expression and release of TAA/TSA through type 1 (T1SS) secretion systems of *Salmonella Typhimurium* have also been studied [51]. Fensterle et al. showed that mice immunized with an *S. Typhimurium* strain release prostate-specific antigen (PSA) via the HlyA (T1SS) system, activate CD8+ T lymphocyte-mediated immune response, and ultimately inhibit tumor development [51,52]. The release of peptides from *Listeria monocytogenes* p60 protein simulates the tumor antigen through T3SS of *S. Typhimurium* in a murine model of fibrosarcoma, demonstrating that 80% of mice immunized with p60 peptide were protected after a fibrosarcoma tumor cell challenge [53,54]. A live strain of *Pseudomonas aeruginosa* has been genetically engineered to transfer *Yersinia (T3SS)* YopE and YopH protein via the T3SS into mammalian cells [55]. This strain generates CTL responses against invading tumors in vivo [55].

### 2.5. Bacterial Mutations 

A variety of rod-shaped bacteria, comprised of both the Gram-positive and Gram-negative groups, have been demonstrated to produce minicells through abnormal cell division. These minicells have the same properties of a normal cell membrane, ribosomes, RNA, and protein, but they lack a bacterial chromosome [50]. By creating mutations in cell division machinery in common rod-shaped bacteria, such as *Escherichia coli* or *Salmonella enterica*, genetically modified minicells have been loaded with chemotherapeutic drugs [56]. Minicells remain an important potential advancement to drug delivery, primarily because they are unable to proliferate, yet retain virulence properties essential for tumor targeting. 

On the other hand, gene transfer properties of bacteria have played an important role in their potential for therapeutic drug delivery. Studies have shown that intracellular bacteria are known to transfer genes to mammalian cells in both in vitro as well as in vivo settings. Several different kinds of bacteria have been studied and manipulated for their potential as gene delivery vectors, such as invasive *E. coli*, *Shigella*, *Listeria*, *Pseudomonas*, and *Salmonella*. Gene transfer occurs when attenuated bacteria release plasmid DNA into the cytoplasm of the host cells, which then culminates in the expression of the transfected genes at the cellular level [57]. This can be further targeted for silencing of genes that favor tumor growth through the use of RNA interference. This entails the transfer of small hairpin RNAs (shRNAs) encoded into a plasmid, which are then transfected in the cytoplasm into small interfering RNAs (siRNAs), and finally act to promote degradation of target mRNA in tumors. This process has been studied to some degree in *Listeria monocytogenes* and *S. enterica* ssp. Typhimurium-expressing targets, such as CTNNB1, Stat3, or Bcl2, all of which are implicated in tumor survival [56].

## 3. Pathogenic and Non-Pathogenic Bacteria in BBCT

These bacterial-based mechanistic strategies have been extensively studied using various pathogenic and non-pathogenic bacteria-targeting BBCT (Table 1). 

### 3.1. Pathogenic Bacteria in BBCT

#### 3.1.1. *Salmonella* spp.

*Salmonella enterica serovar Typhimurium (S. typhimurium)* is a Gram-negative bacterium that is responsible for causing gastroenteritis in humans [58]. *S. typhimurium* is known to be one of the most promising bacterial mediators of cancer immunotherapy, which can be easily manipulated. Thus, it has been engineered and designed in many studies that explore the bacterium as a cancer-targeting therapeutic. *S. typhimurium* has been further investigated in combination with other classical treatments such as chemotherapy or radiotherapy as a synergistic treatment within the tumor microenvironment [59]. *S. typhimurium* is also a popular target due to its ability to grow in both aerobic and anaerobic environments, and, therefore, able to colonize in both non-hypoxic and hypoxic tumors [60]. Salmonella has shown promise due to its ability to specifically proliferate at tumor sites [9]. While Salmonella is often used in cancer therapy for its immunostimulant effects, applications for its use as a therapeutic delivery vehicle are plentiful. For example, Loeffler et al. genetically engineered *S. typhimurium* to express either the proapoptotic Fas ligand or CCL21, a chemokine with anti-tumor properties, and utilization of both proteins has shown inhibition of primary tumors and reduction in metastases in in vivo breast cancer models [61]. Further, *S. typhimurium* has been engineered to produce TNF-related, apoptosis-inducing ligand (TRAIL) controlled by a prokaryotic radiation-inducible promoter, recA. As a natural inducer of apoptosis and tumor cell death, TRAIL is a desirable cytokine to be secreted as a cancer therapy. In vivo results from this model have revealed inhibition of mammary tumor growth and substantially increased rates of survival [56,62]. Other genes, such as cytolysin (HlyE), have been successfully expressed in *S. enterica sv. Typhimurium* under the regulation of a hypoxia-inducible promoter. As a pore-forming toxin, cytolysin has been shown to be effective in murine mammary tumors when specifically targeted to hypoxic regions [9,56]. Yoon et al. investigated the possible anti-tumor properties of TNF-α encased in a Salmonella capsule. TNF-α is a well-known inflammatory factor and promoter of cancer, and, in this study, exhibited anti-tumor effects when assessed in an in vivo model of triple-negative breast cancer (TNBC) [63]. The positive results of these earlier studies have led researchers to further investigate the treatment delivery potential of Salmonella more extensively in animal models.

In their study, Li et al. showed that plasmids co-expressing ENDO-VEGI151 and survivin siRNA have successfully been transferred into an attenuated strain, *S. typhimurium* SL7207. Both genes show promise for use in cancer treatment, as survivin is an apoptosis inhibitor and ENDO-VEGI151 is a promoter of anti-angiogenesis. This treatment demonstrated an inhibition rate of over 90% in a mouse model with a xenografted human cancer tumor [64]. However, these are not the only genes that have been explored in animal experiments utilizing *S. typhimurium*. In the 4T1 TNBC mouse model, *S. typhimurium* was manipulated to express and secrete TGFα-PE38, a potent immunotoxin. There are several components to TGFα-PE38, including transforming growth factor alpha (TGFα) and epidermal growth factor receptor (EGFR). PE38 has been used for its general cytotoxic properties; however, EGFR is specific to treatment in cancer due to the finding that approximately half of TNBCs and inflammatory breast cancers (BCs) overexpress this gene, demonstrating inhibition in the growth of solid tumors [65]. In a recent publication, Mansour et al. used *Salmonella typhimurium* VNP-20009 (VNP) to deliver polypeptide Laz, which is inherent to the Neisserial group of bacteria. Polypeptide Laz crosses the blood–brain barrier during Neisserial infections. However, in this study authors limited their system to express the therapeutic protein Laz at the hypoxic tumor region under hypoxia-induced promoter, HIP-1, selecting Laz upstream for targeting hypoxic tumors [66].

A Salmonella strain (KST0650) was developed as an oxytolerant-attenuated variant from the parental strain KST0649 (ΔptsIΔcrr), via the application of radiation mutation technology (RMT). This newly developed strain was shown to have a 20-times-higher replication rate in cancer cells lines (CT26) and was comparatively less virulent than the parental strain KST0649 [67].

While Salmonella has a plethora of characteristics desirable for targeted cancer treatment delivery, there are many considerations to keep in mind due to the major role of Salmonella in food-borne illnesses, and, thus, the fear triggered by it. While technology has advanced to the point that we now understand bacteria enough to manipulate and attenuate them for alternative uses, there is still much left to be understood and uncovered about the role of Salmonella in cancer treatment before it can become a mainstay in therapy.

#### 3.1.2. *Escherichia* spp.

*Escherichia coli* (*E. coli*) has been engineered and utilized for a variety of uses in science and medicine, with cancer treatment being no exception. Like Salmonella, intravenously administered *E. coli* has been proven to have the ability to target and colonize hypoxic regions of tumors. Genetically engineered *E. coli* strain K-12 secretes cytolysin A (ClyA) and has been administered as a single treatment intravenously in mice with CT26 colon carcinoma, 4T1 metastasizing TNBC, and B16 melanoma tumors [68]. *E. coli* and *S. enterica* are known to produce hemolytic protein ClyA, a 34 KD protein, which induces apoptosis through its pore-forming activity. In this study, administration of *E. coli K-12*-expressing ClyA significantly decreased tumor growth rates initially, but, later, tumor growth progressed. This outcome may be improved by providing subsequent doses of treatment or combining it with other therapies. *E. coli* was further used as a surrogate to produce another bacterial toxin, a pore-forming protein (α-hemolysin) gene from *Staphylococcus aureus* (SA). Within 24 h, α-hemolysin was released and resulted in 93% cell death, with 4T1 tumor volume reduced to only 9% viable tissue [8]. While these studies are promising, research is still just brushing the surface on the implications of *E. coli* in drug delivery. Chiang et. al. have shown the role of bio butyrate in bacterial cancer therapy, by metabolically engineering Escherichia coli 1917 (EcN) to synthesize butyrate, resulting in the EcN-BUT strain [69].

More recently, *E. coli* has been reassessed in cancer treatment in a variety of breast and other cancer models. In 2018, Zhang et al. looked at the use of *E. coli* Nissle 1917 (EcN), because of its known ability to infiltrate the barrier of tumors and replicate in the tumor area, which is between necrotic and viable tissue [70], to produce minicells. Here, the minicells were loaded with doxorubicin, a common chemotherapy drug that inhibits cancer cell division by blocking the enzyme topoisomerase. Additionally, EcN was manipulated to display pHLIP, an insertion peptide used to deliver chemotherapeutic drugs without the need for further modification. Minicells were shown to effectively kill MCF7 and 4T1 cells in vitro and further were shown to be successful in the penetration of hypoxic and necrotic tumor tissue in mice challenged with 4T1 cells. In other work, *E. coli* has been engineered to release a single-domain antibody (nanobody) targeting CD47 within the tumor. CD47, alternatively known as integrin-associated protein (IAP), is a transmembrane protein with many functions, one of which is to help dispose of diseased or aged cells. This treatment was utilized in several in vivo cancer models, including 4T1 TNBC, B16 melanoma, and A20 murine lymphoma, and was shown to reduce rapid tumor progression and increase levels of tumor-infiltrating T cells [71]. 

#### 3.1.3. *Listeria* spp.

One of the most popular vectors for cancer immunotherapy is Listeria monocytogenes, a Gram-positive, facultative anaerobic bacterium. Listeria is mostly known for its association to food-borne illness; however, many of the characteristics that make Listeria pathogenic are the same ones that are now being engineered for use as delivery systems in cancer therapy [72]. Listeria can hijack mechanisms of the host cell cytoskeleton to remain intracellularly mobile and spread from cell to cell [73]. It has been suggested that the use of Listeria may allow treatments to migrate deeper into tumors than with other microbial species, possibly due to their innate ability to evade the phagolysosome and assist in the delivery of plasmid DNA into the cytoplasm [56,74]. Listeria has been engineered in a variety of ways to achieve this end goal, including the early investigation of *L. monocytogenes* paired with nanoparticles, which were shown to effectively express GFP in solid human tumors [75]. Their tumor-targeting properties were demonstrated in in vivo tumors, where *L. monocytogenes* was shown to invade and proliferate in tumors, to ultimately deliver therapeutic genes [76]. Understanding this potential, *L. monocytogenes* was then paired with tumor-associated antigens (TAAs) for enhanced specificity, such as MAGE-B, which is of particular interest in breast cancer because of its frequency of expression in human breast cancer biopsies. This has also been assessed in 4T1 TNBC, where it is confirmed that MAGE-B treatment reduces metastases and promotes tumor cell death in vivo [77,78].

While Listeria possesses several characteristics with potential benefit, one of the most important to note is the presence of the pore-forming protein, listeriolysin O (LLO). LLO helps to ensure the transit of DNA molecules from endosomes into the cytoplasm of target cells. The effectiveness of LLO in relation to drug delivery has been examined in various manners. This has been used in a two-component system, where a neutral HER2-targeting liposome is attached to LLO, combined with condensed plasmid DNA with cationic polyethylene glycol (PEG) and modified polylysine (PL/DNA). When targeted to an endosome, LLO is able to disturb the integrity of the endosome, resulting in cytoplasmic delivery and expression of plasmid DNA. Ultimately, this culminates in increased expression within HER2-positive breast cancer cells lines [79]. Alternatively, polylacticcoglycolic acid (PLGA) microspheres have been incorporated with LLO to optimize cytosolic delivery to target cells and subsequent presentation to the immune system [80]. It has been shown that phagocytic cells readily take up the combination of microspheres with LLO, which consequently results in an increase in peptide-MHC-I expression on the cell surface. Additionally, cytotoxic T cells have been stimulated through the activation of a T hybridoma cell line through treatment with microspheres and LLO. 

Further, LLO has been used with specific anti-tumor therapies to evaluate efficacy in cancer treatment. In one study, LLO was linked to a luciferase-encoding PEGylated polylysine core disulfide in combination with the monoclonal antibody, trastuzumab. In this system, LLO was necessary to establish transit of DNA molecules into the cytoplasm while trastuzumab allowed targeting of HER2 receptors in breast cancer. Treatment in MCF7 and MCF7/Her18 breast cancer cell lines demonstrated increased expression of luciferase activity, indicating successful gene delivery into tumor cells [81]. More recently, Listeria has been investigated as a possible source for the generation of drug-delivering nanoparticles. Functional nanoparticles have been produced from self-assembling *Listeria innocua* DNA binding protein (LiDps) in starved cells, and then further manipulated with the addition of Gaussia princeps luciferase and Zinc(II)-protoporphyrin IX (ZnPP). Tumorigenic cells have shown effective uptake of Gluc-LiDps-ZnPP conjugate, which acts against tumors by producing reactive oxygen species through Bioluminescence Resonance Energy Transfer (BRET). Ultimately, this resulted in significant suppression in the migration of surviving SKBR3 breast cancer cells [82]. Through advancements in the manipulation of Listeria, this bacterium has become a favorite candidate in the quest for more effective treatment delivery systems.

#### 3.1.4. *Clostridium* spp.

Clostridium is known to be one of the largest prokaryotic genera, comprised of anaerobic spore-forming bacteria. The Clostridium group of bacteria resist harsh environmental conditions such as high temperature and dehydration by producing endospores [83]. Clostridium also presents as a desirable delivery vehicle for therapeutic cancer drugs, having a natural ability to seek out and prosper in low-oxygen environments, such as those experienced in the core of the tumor microenvironment [9]. Clostridium is limited to tumor sites due to its inability to survive in other normal tissues that are rich with oxygen [70]. Clostridium and its related spores have been heavily implicated in cancer immunotherapy, with drug delivery potential taking lower precedence. The prevalence of clostridial spores in cancer therapies is well studied and has been reviewed in a number of scientific publications [84,85,86,87,88]. Various subtypes of Clostridium have been tested as anti-cancer agents including *C. butyricum*, *C. tetani*, *C. histolyticum* [89,90], *C. beijerinckii* [91], and *C. acetobutylicum* [92]. *Clostridium acetobutyicum* was one of the first to be investigated for its anti-cancer activity, as studies showed the ability to effectively engineer *C. acetobutyicum* to secrete mouse TNF-α. Similarly, *C. acetobutyicum* was also shown to be able to successfully secrete interleukin-2 (IL2), which, in humans, is known to stimulate immune cells by promoting the development of T cells [93]. 

*Another Clostridium member, Clostridium novyi,* has been genetically modified by removing a residential phage-carrying α-toxin to make the strain non-pathogenic. This major toxin was shown to be responsible for the toxicity of *C. novyi* by Vogelstein et al., who studied 26 anaerobes for their capability to divide and disseminate in a human colorectal cancer xenograft model [4]. Vogelstein et al. also developed the strain *C. novyi*-NT, which has been examined as an alternative for cancer immunotherapy and is undergoing a Phase I clinical study for the treatment of refractory tumors (NCT01924689). *C. novyi*-NT induces a vigorous inflammatory response engaging pro-inflammatory cytokines such as MIP-2, IL-6, G-CSF, and TIMP-1, which employ a significant number of immune cells to the site of infection and tend to increase long-lasting, adaptive anti-tumor immunity [94,95,96]. The precise mechanisms by which *C. novyi*-NT mediates tumor elimination are unknown; however, one of the major observations indicates that administration of *Clostridium difficile* (*C. diff*.) toxin B (TcdB)-treated CT26 colon cancer cells and B16–F10 melanoma cells in mice results in an extended tumor-specific immune response, providing insight into possible mechanisms for anti-tumor responses from *C. novyi*-NT [97].

With promising applications as a hypoxia-targeted delivery system, Clostridium deserves to be further investigated in this era of improved biotechnological methods. Clostridium-directed antibody therapy (CDAT) is another area of interest where Clostridium is mutated to induce production of high-specificity antibodies. By heterologous gene transfer, *C. novyi*-NT has been introduced with a heavy-chain subclass of antibodies (VHH), specifically VHH targeted against HIF1α [98]. These antibodies, when expressed in mammalian cells, inhibit HIF activity. Independent responses from *C. novyi*-NT are moderately rare; however, *C. novyi*-NT can be combined with other chemotherapeutic agents or radiation, a technique known as Combination Bacteriolytic Therapy (COBALT). The main reason for using *C. novyi* with other therapies is that *C. novyi-*NT can reach the necrotic and hypoxic region of the solid tumors, which are conventionally known to be resistant to other treatments such as radiation and chemotherapy. While *C. novyi*-NT has tremendous potential as a cancer therapeutic, numerous challenges still remain to be resolved before Clostridium-based BBCT can obtain essential regulatory approval and can be applied in the clinic. 

#### 3.1.5. *Corynebacterium* spp.

*Corynebacterium diphtheriae* is a group of Gram-positive bacteria and the causative agent of diphtheria. Corynebacterium can grow either as an aerobic or as a facultative anaerobic [99]. Diphtheria toxin (DT) is a highly robust toxin, and simply the entry of a single molecule into a cell can be toxic [99]. Due to this high toxicity, DT has been extensively studied to treat cancer cells by genetically deleting the cell receptor-binding domain and re-arranging the catalytic portion with the targeted proteins that collectively bind to the surface of the targeted cancer cells. DT-based immunotoxin (DTAT) can perform anti-tumor actions against different types of cancers, including glioblastoma and pancreatic cancer, through urokinase receptors (uPARs). A series of in vitro and in vivo studies have been used to show the ability and anti-tumor effects of immunotoxins (DTAT, DTAT13, and DTATEGF), which are directed by uPAR [100]. Although most of the pre-clinical work has shown positive responses, there remain no known clinical trials or possible clinical evaluations for any uPAR-based immunotoxins [100]. Not only the full length but also the truncated versions of DT have been used to establish recombinant immunotoxins against a series of cancers [101]. Various other DT-based immunotoxins have also been studied, which are specifically targeted to cancers of interest, including cell-penetrating peptide BR2 and receptor of Treg cells, CCR4 [102], DT386-BR2 [103], and DT-anti-CCR4 [104]. A genetically modified fusion protein, *Denileukin diftitox* (Ontak), was generated by fusing IL-2 and diphtheria toxin. This toxin introduces diphtheria toxin into the targeted cells that highly express IL-2 receptors, which hinders protein synthesis, thus causing cell death [105]. Ontak is also the first known immunotoxin approved by the Food and Drug Administration (FDA) for the treatment of cutaneous T cell lymphoma (CTCL) [106]. However, production of Ontak was suspended in early 2011 due to issues in preparation of Ontak and reports of the presence of contaminants, along with adverse events (AEs) [107].

#### 3.1.6. *Pseudomonas* spp.

*Pseudomonas aeruginosa* is a Gram-negative, aerobic bacterium, which, under certain environmental conditions, can also grow as a facultative anaerobic bacterium [108]. Pseudomonas is known to have a plethora of virulence factors including toxins, which play a key role in its pathogenesis, including phytotoxic factor, pigments, hydrocyanic acid, proteolytic enzymes, endotoxins, and exotoxins [109]. Pseudomonas exotoxin A (PE) is known to be one of the major toxic virulence factors of pseudomonas [110] and has been extensively studied for its anti-tumor specificity [111,112] by inhibition of eukaryotic elongation factor 2 (Eef2) activity [111]. The ADP-ribosylation activity of pseudomonas affects the protein synthesis of the infected host cells. Various molecular strategies have been used by PE for effective killing of the host cell. Immunotoxins derived from PE have been tested in various pre-clinical as well as clinical studies against a variety of hematologic malignancies and solid tumors with promising results. In a clinical trial, Moxetumomab pasudotox, an anti-CD22 immunotoxin agent, was used for the treatment of adults with relapsed or refractory hairy cell leukemia (HCL). Eighty patients were treated and 41% of the patients demonstrated complete remission [108]. 

In other studies, the immunotoxin, SS1P, targeting mesothelin has been administrated in combination with known immune-modulating chemotherapeutic agents, including pentostatin and cyclophosphamide to mesothelioma patients [112]. *Pseudomonas* spp. has also been genetically engineered to be used as delivery vehicle. *Pseudomonas aeruginosa-*mannose-sensitive hemagglutinin (PA-MSHA) has been engineered to attach mannose-sensitive fimbriae type 1 onto its surface. This strain has shown anti-cancer cytotoxic activities against breast, lung, cervical, hepatocellular, colon, and pancreatic cancer cell lines [113,114]. 

### 3.2. Non-Pathogenic Bacteria/Probiotics in BBCT

#### 3.2.1. Lactic Acid Bacteria (LAB)

Lactic acid bacteria (LAB) are fastidious, Gram-positive, non-spore-forming cocci or rods, known to have high tolerance to low pH [115]. LAB are known to be beneficial not only for the balance of intestinal flora but also for their antimicrobial, antioxidant, anti-inflammatory, and anti-cancer effects [116]. Lactobacillus, Lactococcus, Streptococcus, Leuconostoc, Oenococcus, and Pediococcus [117] together form a heterogenous group of bacteria under the genera of LAB. LAB have proven to be a safe and appealing option in the realm of potential bacteria for use as drug delivery systems. LAB have an extensive history of being safe for human use in various areas of medicine and food, and now studies have implicated them in the distribution of drugs to solid tumors [118]. LAB inhabit the small and large intestines of humans and animals, and have been shown to have the capacity to travel after IV administration to solid tumors, where they can accumulate and proliferate [28]. The development of biotechnological tools has allowed progression to a point where these organisms can be engineered to secrete a protein of interest into the extracellular tumor environment to provide a more targeted therapeutic benefit. LAB strains show strong antioxidant properties due to their high catalase activity and α, α-diphenyl-β-picrylhydrazyl (DPPH) free radical scavenging activity. LAB also profoundly catalyze anti-inflammatory activity by the activation of anti-inflammatory cytokines (e.g., IL-10) and decreased expression of pro-inflammatory cytokines (e.g., IL-6) [119]. Numerous studies have depicted that probiotics reduce colorectal cancer-associated bacteria such as Fusobacterium and peptostreptococcus [120]. Regular intake of LAB has been shown to reduce breast cancer risk in women [121]. Fermented food containing L. acidophilus, L. bulgaricus, Streptococcus lactis, or Bifidobacteria have been shown to inhibit the proliferation of ER+ breast cancer in an animal model [122,123,124]. Thus far, the most common LAB currently used as drug delivery vehicles are Lactobacillus, Lactococcus, and Bifidobacterium species.

#### 3.2.2. *Lactobacillus* spp. 

Lactobacillus is a genus of Gram-positive, rod-shaped bacteria inhabiting the intestinal microbiome of humans and other mammals. As one of the major probiotic bacterium in the intestine, the key role of this bacterium is to share lactic acid fermentation with other bacteria and further strengthen the intestinal barrier [125]. *Lactobacillus plantarum* (L. *plantarum*) is being studied for various clinical applications, including cancer treatment. The L-14 form of *L. plantarum* extract has been shown to inhibit the viability and relocation of A375 cells, as well as regulating the expression of genes involved in migration in a human malignant melanoma model [126]. *Lactobacillus casei* harbors anti-tumor effects mediated by the downregulation of IL-22 and upregulation of caspases, inducing apoptosis [127]. Lactobacillus targets malignant cells by producing bacteriocins such as nisin that induce apoptosis and reduce cell proliferation by cell cycle arrest in the G2 phase [128]. Kim et al. reported using probiotic *Lactobacillus kimchicus* DCY51 for non-covalent loading of ginsenoside compound K (CK). CK is highly regarded in traditional Chinese herbal medicine due to its bioactive triterpenoid saponins, and it has been shown to inhibit hormone-independent breast cancer by downregulating cyclin D1, an important part of the G1 phase of the cell cycle [78]. This study demonstrated that nanoparticle-bound DCY51 kills more A549 cells (human lung adenocarcinoma cell line) and HT29 cells (human colorectal adenocarcinoma cell line) compared to ginsenoside CK treatment alone [78]. Another study using a melanoma mouse model suggested that strain *L. reuteri* FLRE5K induces higher levels of the cytokines TNF-α and IFN-γ, which stimulate immunity and interfere with proliferation of melanoma cells [129]. Alternatively, Lactobacillus enriched with selenium has shown positive anti-tumor effects, as LAB can form elemental selenium nanoparticles (SeNPs) by reducing selenium ions and then proceeding to drop off the nanoparticles intracellularly. As an anti-carcinogenic essential micronutrient, selenium acts by preventing activation of oncogenes, and, therefore, preventing the transformation of normal cells into malignant ones. This was evaluated in mice bearing 4T1 breast cancer, where treatment with enriched Lactobacillus was shown to increase survival and decrease the number of tumor metastases to the liver [130]. In another study, fluorescent inorganic cadmium sulfide (CdS) nanoparticles were successfully transported into MCF-7 breast cancer cells using *Lactobacillus* spp. as a vector [131]. In a titration study, the authors showed that increasing concentrations of CdS NPs gradually decreases the metabolic activity of MCF-7 cells until a peak concentration of 5 ppm CdS NPs is reached, with 80% cell death at 24 h and complete cell death at 48 h [131]. 

#### 3.2.3. *Lactococcus* spp. 

Lactococcus was first employed as a delivery vehicle for drugs in diseases other than cancer, such as inflammatory bowel disease, where it has been genetically modified to secrete IL-10 [132]. Following this approach to other diseases, studies have followed suit by exploring Lactococcus as a rising form of drug and gene delivery in various types of cancer. *Lactococcus lactis* has been used as a KiSS1 peptide-producing factory, where *L. lactis* NZ9000-401 was constructed to express human KiSS1. KiSS1 peptide plays an important part as a tumor suppressor inhibiting cancer metastasis [70]. HT-29 cells displayed morphological changes and apoptosis when treated with *L. lactis-*expressing KiSS1. While this study looked at the effects in HT-29 human colon cancer cells, KiSS1 is expressed in human breast cancer, leading to the conclusion that this therapy poses an opportunity to benefit breast cancer treatment specifically. Alternatively, the usefulness of *L. lactis* in breast cancer has been proven by its success in secreting drugs already shown to be effective in reducing tumor size and slowing growth. An active form of *L. lactis* has demonstrated successful expression of Mig and IP-10 [133], both of which are chemokines and function to draw immune cells to the site of active infection. Both Mig and IP-10 have anti-angiogenic properties critical for tumor immunity. Additionally, *L. lactis* has been genetically engineered for induction of IL-12, a member of an interleukin family with immunoregulatory effects, including stimulation of IFN secretion and Th1 immune responses, as well as inhibition of Th2 responses. While Lactobacillus holds promise as a safe and effective delivery vehicle for breast cancer therapy, there is still much left to learn and assess before it will be deployed to the forefront of cancer treatment.

#### 3.2.4. *Bifidobacterium* spp. 

Bifidobacterium spp. is a branched, non-motile, obligate anaerobic bacteria. It is among the first bacteria to be colonized in the human gut [134]. Among 50 known species of Bifidobacterium spp. in various environments, only 10 are found in humans. Many studies have reported using Bifidobacterium spp. for its anti-tumor activities [135]. Although these microorganisms successfully colonize tumor cells, anti-tumor effects of Bifidobacterium have not yet been fully determined. However, preliminary observations have led to the investigation of *Bifidobacterium* spp. as a major delivery vehicle that can be bioengineered and modified to express genes of interest for cancer immunotherapy. Wang et al. showed that genetically engineered *B. breve,* modified to express IL-24 (*B. breve*-IL24), inhibits head and neck tumor growth by inducing apoptosis [136]. It has been demonstrated in mouse models that bioengineered Bifidobacterium spp. could deliver enterolactone, which converts fatty acids into pectin oligosaccharides (POS), which significantly delay the development of leukemia [137]. The use of Bifidobacterium for gene delivery was first investigated by determining whether *B. longum* 105-A transformed with a spectinomycin-resistant gene effectively transfers resistance in mice, thereby indicating gene transfer [28]. In later years, this concept was applied to cancer-specific gene therapies such as the endostatin gene, an inhibitor of basic fibroblast growth factor (bFGF)-stimulated vessel endothelial cell proliferation. A dormant state in primary tumors can be achieved through systemic administration of endostatin in tumor-bearing mice. Endostatin-carrying *B. adolescentis* has demonstrated inhibition of hypoxic tumor growth and angiogenesis when administered intravenously to mice infected with Heps liver cancer [138]. 

Since it was established that Bifidobacterium could be used as a relatively safe and competent vehicle for treatment delivery, studies assessing specific cancer therapies have been conducted. The production of the enzyme, cytosine deaminase (CD), by *B. longum* has been combined with 5-fluorocytosine (5FC) in solid tumors, including breast cancer. A resultant high concentration of 5-fluorouracil (5FU) was found to be localized in the tumor, because of the reaction between 5FC and CD. This turned out to be beneficial for cancer therapy as 5FU is a more toxic prodrug than 5FC; therefore, it is possible to refrain from systemically distributing 5FU and instead using 5FC, which will only be converted to 5FU at the tumor site [118,139]. Bifidobacterium has also demonstrated efficacy in solid tumors when orally administered, making it of particular interest. *B. breve* has been shown to be able to successfully colonize solid B16 murine melanoma tumors after being orally administered and translocated to the gastrointestinal tract [140]. However, Bifidobacterium has primarily been evaluated through intravenous injection, with more common therapeutic breast cancer drugs making their way into the literature. Trastuzumab has become a mainstay treatment against HER2-positive breast cancer as a HER2-targeting monoclonal antibody. A genetically engineered version of *B. longum* has displayed significant repression of xenographed human HER2-positive tumors in mice [141]. Furthermore, effective delivery to solid tumors using Bifidobacterium microbots was demonstrated in a mouse model through fluorescent imaging of CdSeS quantum dots [131]. 

#### 3.2.5. *Magnetococcus* spp.

In recent years, there has been an increased interest in exploring environmental microorganisms for properties with potential applications in cancer therapy. Found in the sediment in the depths of the water, a group of anaerobic bacteria known to align themselves with the planet’s geomagnetic field, magnetotactic bacteria, have come to light as a possible beneficial tool for drug delivery [142]. These properties necessary to target tumors, as magnetotaxis, in addition to flagellar motors, are what allow the bacteria to migrate to and reside in low-oxygen areas [143]. The magnetic properties of these bacteria are also helpful in targeting tumors because they make it possible to magnetically guide them to the site of the tumor, in addition to their natural hypoxia-seeking state. *Magnetococcus marinus* MC1 is currently the most tested magnetotatic bacteria in the delivery of cancer therapeutics [7]. This Gram-negative coccus was found in the Atlantic Ocean and has been studied by covalently binding drug-containing nanoliposomes. Accordingly, MC-1 cells bearing nanoliposomes were injected near the tumor site and guided magnetically. This resulted in up to 55% of MC1 entering hypoxic tumor areas of SCID Beige mice within HCT116 colorectal xenographs [144]. Based on early successes, magnetotactic bacteria are a notable development agent in drug delivery using microorganisms and needs to be further explored and applied to more extensive in vivo tumor testing.

**Table 1:** Bacteria for cancer immunotherapy.

Bacteria	Strain	Mutated/Gene Modified	Cancer Type	Phenotypic Description	Ref
Pathogenic bacteria-mediated cancer immunotherapy					
*Salmonella typhimurium*	A1-R	∆leu/∆arg	Prostate cancer	Auxotrophic strain defective in synthesis of leucine and arginine	[145]
	VNP20009	∆msbB/∆purI	Metastatic melanoma, Glioblastoma, Pancreatic cancer, Colon cancer, Breast cancer	Modification of Lipid A structure; reduced ability to induce TNF-α secretion; deficiency in adenine synthesis	[146,147,148]
	SHJ2037	∆relA/∆spoT		∆ppGpp (global regulator); reduction in bacterial invasion	[149,150]
	SL3261, SL7207, BRD509, YB1	aro-	Prostate cancer, Melanoma, Breast cancer	Mutations in aromatic amino acid biosynthesis	[19,151,152,153,154]
	LH430; VNP (Pho/Q-)	∆phoP/∆phoQ	Colorectal cancer, Renal cancer	Reduced bacterial survival in macrophages	[37,155]
	MvP728	∆purD/∆htrA	Colon carcinoma, DBT glioblastoma, Melanoma	Defective in purine biosynthesis, produces heat-shock protein response to stress stimuli	[156]
	YB1; ST8	∆asd	Breast cancer, Colon cancer	Defective in diaminopimelic acid (DAP) synthesis	[19,157]
	X4550	∆cya/∆crp	Osteosarcoma	Disabled production of cAMP (cyclic adenosine monophosphate) synthetase and cAMP receptor protein	[158]
	RE88	∆dam	Breast carcinoma	Defective in DNA adenine methylase production	[159]
	SB824	∆sptP	Melanoma	Defective in pathogenicity island 1 (SPI-1)	[160]
	ST8	∆gmd	Colon cancer	Unable to replicate beyond the anaerobic regions of tumors	[137]
	SF100; SF200; S364	rfa-, ΔpagP/ΔpagL/ΔlpxR	Colorectal cancer, Fibrosarcoma	Highly truncated LPS and attenuated bacterial virulence	[161,162]
	MPO378	∆purD/∆upp	Breast Cancer cell line	Inefficient in purine biosynthesis and uracil phosphoribosyl transferase	[162]
	FlaB	*Vibrio vulnificus*flagellin B	Colon cancer	Engineered FlaB from Vibrio vulnificus-secreting bacteria	[150]
*Listeria monocytogenes*	rLM	Lm-LLO-E7	Cervical cancer, Leukemia, Ovarian cancer, Prostate cancer, Colon cancer, Breast cancer	Secretes a fusion protein comprised of nonfunctional LLO joined with HPV protein E7	[163]
	XFL7	Lm-LLO-PSA	Prostate cancer	Significantly higher number of IFN-γ-secreting cells	[164]
	DP-L4029	∆actA	Colon cancer, Lung cancer	Defective surface-bound ActA polypeptide, constitutes LLO activity at physiologic pH	[44,165,166]
	DP-L4017	LLO L46IT, LLOD26	Lung cancer	Cytotoxic, defective cell-to-cell spreading and greater percentages of splenic- and tumor-infiltrating, antigen-specific CD8+ lymphocytes	[5,42,167]
	DP-L4042	∆PEST	Colon cancer, Lung cancer	Cytotoxic, defective cell-to-cell spreading	[42,167]
	DP-L4405 DP-L4406	∆inIA/∆niB	Colon cancer	Impaired InIA-mediated infection	[168]
	CS-L0001	∆actA/∆inlB	Colon tumor lung metastases	Defective in cell-to-cell spreading	[44]
	CS-L0002	∆actA/∆lplA		*L. monocytogenes* vaccine vectors expressing influenza A nucleoprotein	[169]
	DP-L4038	∆actA/L461T LLO		Inadequate surface-bound ActA polypeptide, constitutes LLO activity at physiologic pH	[165,166]
*Mycobacterium bovis*	BCG Pasteur	1137P2	Bladder cancer	Cancer cell phagocytosis by increasing proinflammatory cytokine activation and immune system	[47,170,171]
*Clostridium novyi*	NT	∆toxA/∆toxB	Glioblastomas neuroshphere, Colon cancer	Produces specific enzymes and toxins capable of destroying cancer cells	[172,173,174]
*Escherichia coli*	MG1655		4T1 breast cancer	Optimized physicochemical properties for bacterial attachment; Low cost for bioconjugation	[175]
*Streptococcus pyogenes*	OK-432		Lymphangioma intraoral ranula	Including TNF, IL-8, IL-6, IFN-γ, and VEGF; increase in WBCs	[176,177,178,179]
*Pseudomonas aeruginosa*	*F10*		Lung cancer, Breast cancer, Cervical cancer, and Colon cancer	Anti-tumor effects of 2,4-diacetylphloroglucinol (DAPG) extracted	[180]
	(PA-MSHA)		Pancreatic cancer	Anti-tumor effect of *P. aeruginosa*-MSHA (mannose-sensitive hemagglutinin) inducing apoptosis by the EGFRa pathway and caspase signaling	[181]
			Hepatocellular carcinoma	Anti-tumor effect of *P. aeruginosa*-MSHA (mannose-sensitive hemagglutinin) by EGFR/Akt/IκBβ/NF-κB pathway	[182]
Non-pathogenic bacteria-mediated cancer immunotherapy					
*Lactobacillus reuteri*	PTCC 1655	WT	Gastric cancer	Probiotic-based strategies: inhibition of cell proliferation by downregulation of uPA/uPA receptors (uPARs)	[183]
	FLRE5K1	WT	Melanoma	Preventive effect of *L. reuteri* on melanoma	[129]
*Lactobacillus plantarum*		WT	Colon cancer, Breast cancer, Oral cancer	Produces antioxidants, increases TNF-α, induces caspase-3 activity, inactivates Wnt/β-catenin signaling	[184,185]
*Lactobacillus rhamnosus*	SHA111; SHA112; SHA113	WT	Colorectal cancer, Cervical adenocarcinoma, Breast cancer	Apoptosis via up-regulation of BAD, BAX, Caspase3, Caspase8, and Caspase9, and down-regulation of BCL-2 genes	[183]
*Lactococcus lactis*			Head and neck tumor	Anti-tumor effect of nisin: by induction of apoptosis through a calpain-dependent pathway	[128]
*Bifidobacterium bifidum*			Lung cancer	Induction of immune responses, which leads to inhibition of tumor growth by activation of IL-12 and IFN-γ, lymphocyte proliferation, and CD8+ cytolytic induction	[186]
	CGMCC 15068		Colon cancer	*B. bifidum* growth in intestinal health by modulating dysbiosis and the gut metabolic profile	[187]
*Bifidobacterium longum*	NCC2705	WT	Colon adenocarcinoma	*B. longum* as a vector of tumstatin (Tum) inducing significant anti-tumor effect	[137]
	420 and 440	WT	Prostate cancer	*B. longum*-based vaccine inducing immune response against Wilms tumor 1 (WT1) antigen	[188]
*Bifidobacterium breve*	UCC2003	WT	Head and neck tumor	*Strain* expressing IL-24 gene: Apoptosis induction leads to anti-tumor activity	[136]

## 4. Clinical Trials Using Bacteria as Delivery Vehicles

In 1891, William B. Coley used live infections of a combination of *Streptococcus Pyogenes* and *Serratia marcescens* as an immunotherapy against sarcoma [1,189]. Since then, a multitude of bacterial strains have been studied and are now selected for testing in patients in clinical trials (Table 2).

The information gathered in the clinical data shown in the table reveals many major obstacles as well as challenges that need to be addressed for further successful human application of bacteria as cancer immunotherapy in near future. While bacteria alone may not offer the best solution, broadening our knowledge and understanding as well as altering bacteria to have fewer side effects along with anti-tumor agents, immunogenic agents, and/or anti-oncogenes can prove to be beneficial.

## 5. Current Challenges

Due to the advancement of the microbiome as a major player in our search for remission of various human diseases, bacteria as a therapeutic prospect are gaining significant interest in many medical fields [195]. Tumor targeting-bacteria have peculiar distinctive features including unique gene packaging, targeting the hypoxic environment of tumor, and tumor selectivity, which make them an ideal vehicle for delivering therapeutic cargo specifically targeting cancers of various origins. However, although engineered bacteria have gained high therapeutic potential to target tumors, due to high heterogeneity of cancers at the molecular and histologic levels, a single anti-cancer agent may not be able to achieve cure by itself. Thus, a combinatorial approach may be required to develop a promising anti-cancer therapy.

One of the major concerns in the field of BBCT is the toxicity of bacteria due to associated toxins, which may lead to serious infections, considerable side effects, and even death. Researchers are, therefore, using attenuated and genetically modified strains to overcome these adverse outcomes. Reducing or removing specific virulence factors from bacteria by genetic modifications can also remedy the toxicity associated with using bacteriotherapy. However, it should be noted that there is a tradeoff of reducing virulence and removal of virulence factors and clinical outcomes, as removing virulence of a bacteria can reduce the potency of its anti-cancer affects. It is well documented that bacterial strains manipulated for cancer therapies are sensitive to changes in their virulence factors. Microbe-associated molecular patterns (MAMPs) need additional attention when they are adapting to bacterial strains during cancer therapy. However, it has been previously reported that structural changes in LPS can cause changes in the physiology of bacteria to transform from a virulent strain to a strain with anticancer properties. For example, a change in the structure of lipid A to hexa-acylated lipid A, has led to increased affinity for Toll-like receptor 4 (TLR4), which can induce anti-cancer responses [194]. Another major challenge in this field is the short half-life of the bacterial peptide of protein and unstable DNA. 

One of the major caveats of BBCT is that it is not suitable for patients who have been on certain types of chemotherapy, as these may suppress the immune system to the extent that it cannot sufficiently respond to bacterial colonization. Additionally, live bacterial products can colonize in foreign bodies like artificial heart valves, joint replacements, and implanted medical devices, which may serve as reservoirs for infection. Furthermore, recombinant plasmids carried by bacteria can be mutated, thus changing the fate of anti-tumor action before the cancer cells are penetrated. This can lead to various associated risks, including therapy failure, infection, or death [195]. A major public health concern is the development of multi-drug resistance of many of the bacteria used in BBCT. 

## 6. Future Prospects

Careful manipulation of microbes may very well be the next necessary step to making them a routine part of cancer therapy. Conscientious exploitation of microbial mechanisms for their tumor-targeting properties also proposes major applications as a personalized therapy, as this new level of control can be utilized for each patient’s unique tumor type. The ideal microbial therapy will theoretically combine a non-pathogenic but effective species that will consist of not one but multiple strains selected for their specific targets and then ultimately be combined with effective standard treatments for the best possible efficacy. The hypoxia-honing powers of microbes can be combined with other therapeutic methods to target the remaining tumor regions that are richer in oxygen. The genetic flexibly of microorganisms may truly be their greatest strength, allowing for precise tuning of individualized therapy for maximum cytotoxic effects.

The idea of treating cancer with microbes as delivery vehicles has a long way to go before rising to the popularity of current mainstay therapies. Toxicity issues and cultural stigmas must be addressed before microorganisms will be trusted in the realm of cancer therapy. The field of BBCT is still considered to be quite novel and more scientifically sound studies need to be conducted to overcome the ongoing limitations and side effects associated with bacteriotherapy. However, the potential that BBCT holds is impossible to overlook, with a plethora of promising mechanisms that may be manipulated to target tumors and improve patient outcomes. Despite encouraging in vitro and in vivo results of BBCT, very few studies have led to clinical trials. Therefore, it is obligatory that the scientific and clinical communities begin to design additional clinical trials to investigate and harness the efficacy of BBCT.

## Figures and Tables

**Table 2:** Clinical Trials.

Bacterial Strain	Type of Cancer	Clinical Phase	Identifier No.	Reference (All Links Were Accessed on 16 December 2021)
*C. histolyticum*	Lipoma	I	NCT01613313	https://clinicaltrials.gov/ct2/show/NCT01613313
	Lipoma	I	NCT02249052	https://clinicaltrials.gov/ct2/show/NCT02249052
*C. butyricum M55*	Vascular glioblastoma	I	-	[190]
*C. novyi-NT*	Solid tumor malignancies	I	NCT01924689	[173]
	Colorectal cancer	I	NCT00358397	https://clinicaltrials.gov/ct2/show/NCT00358397
	Solid tumor malignancies	I	NCT01118819	https://clinicaltrials.gov/ct2/show/NCT01118819
	Refractory advanced solid tumors	Ib	NCT03435952	https://clinicaltrials.gov/ct2/show/NCT03435952
*L. monocytogenes*	Cervical cancer	II	-	[191]
	Cervical cancer	III	NCT02853604	https://clinicaltrials.gov/ct2/show/NCT02853604
	Metastatic pancreatic tumors	II	-	[192]
	Malignant epithelial mesothelioma Adenocarcinoma of the pancreas, Non-small cell lung adenocarcinoma of the ovaries	I	NCT00585845	https://clinicaltrials.gov/ct2/show/NCT00585845
	HPV-16 +ve oropharyngeal carcinoma	I	NCT01598792	https://clinicaltrials.gov/ct2/show/NCT01598792
*L. monocytogenes (LADD)*	Prostatic neoplasms (castration resistant)	II	NCT01613313	https://clinicaltrials.gov/ct2/show/NCT02625857
	Non-small cell lung carcinoma	I	NCT02592967	https://clinicaltrials.gov/ct2/show/NCT02592967
*S. typhimurium (χ4550)*	Hepatocellular carcinoma	I	-	[193]
*S. typhimurium* VNP20009	Metastatic melanoma, metastatic renal cell carcinoma	I	-	[15]
*S. typhimurium* VNP20009	Melanoma	I	-	[17]
*S. typhimurium VNP20009 expressin*g TAPET-CD (cytosine deaminase)	Head and neck, and esophageal adenocarcinoma	I	-	[[18]]
*S. typhimurium* VNP20009	Advanced metastatic solid tumors	I	NCT00004216	https://clinicaltrials.gov/ct2/show/NCT00004216
	Solid tumors	I	NCT00006254	https://clinicaltrials.gov/ct2/show/NCT00006254
	Neoplasm metastatic tumor	I	NCT00004988	https://clinicaltrials.gov/ct2/show/NCT00004988
*S. typhimurium expressing IL-2*	Liver cancer	I	NCT01099631	https://clinicaltrials.gov/ct2/show/NCT01099631
*S. typhimurium Ty21a VXM01*	Pancreatic cancer	I	-	[194]
Mixed Bacterial Vaccine	Malignant tumors	I	NCT00623831	https://clinicaltrials.gov/ct2/show/NCT00623831

## Data Availability

Not applicable.

## References

[1] Coley W.B. (1893). The treatment of malignant tumors by repeated inoculations of erysipelas. With a report of ten original cases. Clin. Orthop. Relat Res..

[2] McCarthy E.F. (2006). The toxins of William B. Coley and the treatment of bone and soft-tissue sarcomas. Iowa Orthop. J..

[3] Lin I.Y., Van T.T., Smooker P.M. (2015). Live-Attenuated Bacterial Vectors: Tools for Vaccine and Therapeutic Agent Delivery. Vaccines.

[4] Forbes N.S. (2010). Engineering the perfect (bacterial) cancer therapy. Nat. Rev. Cancer.

[5] Wood L.M., Guirnalda P.D., Seavey M.M., Paterson Y. (2008). Cancer immunotherapy using Listeria monocytogenes and listerial virulence factors. Immunol. Res..

[6] Gardlik R., Fruehauf J.H. (2010). Bacterial vectors and delivery systems in cancer therapy. IDrugs.

[7] Łukasiewicz K., Fol M. (2018). Microorganisms in the Treatment of Cancer: Advantages and Limitations. J. Immunol. Res..

[8] St Jean A.T., Swofford C.A., Panteli J.T., Brentzel Z.J., Forbes N.S. (2014). Bacterial delivery of Staphylococcus aureus α-hemolysin causes regression and necrosis in murine tumors. Mol. Ther..

[9] Ryan R.M., Green J., Williams P.J., Tazzyman S., Hunt S., Harmey J.H., Kehoe S.C., Lewis C.E. (2009). Bacterial delivery of a novel cytolysin to hypoxic areas of solid tumors. Gene Ther..

[10] Kalaora S., Nagler A., Nejman D., Alon M., Barbolin C., Barnea E., Ketelaars S.L.C., Cheng K., Vervier K., Shental N. (2021). Identification of bacteria-derived HLA-bound peptides in melanoma. Nature.

[11] Antonelli A.C., Binyamin A., Hohl T.M., Glickman M.S., Redelman-Sidi G. (2020). Bacterial immunotherapy for cancer induces CD4-dependent tumor-specific immunity through tumor-intrinsic interferon-γ signaling. Proc. Natl. Acad. Sci. USA.

[12] Leventhal D.S., Sokolovska A., Li N., Plescia C., Kolodziej S.A., Gallant C.W., Christmas R., Gao J.-R., James M.J., Abin-Fuentes A. (2020). Immunotherapy with engineered bacteria by targeting the STING pathway for anti-tumor immunity. Nat. Commun..

[13] Miyazaki M., Yuba E., Harada A., Kono K. (2015). Hyaluronic acid derivative-modified liposomes as pH-sensitive anticancer drug delivery system. J. Control. Release.

[14] Chen W., Wang Y., Qin M., Zhang X., Zhang Z., Sun X., Gu Z. (2018). Bacteria-Driven Hypoxia Targeting for Combined Biotherapy and Photothermal Therapy. ACS Nano.

[15] Toso J.F., Gill V.J., Hwu P., Marincola F.M., Restifo N.P., Schwartzentruber D.J., Sherry R.M., Topalian S.L., Yang J.C., Stock F. (2002). Phase I study of the intravenous administration of attenuated Salmonella typhimurium to patients with metastatic melanoma. J. Clin. Oncol..

[16] Fritz S.E., Henson M.S., Greengard E., Winter A.L., Stuebner K.M., Yoon U., Wilk V.L., Borgatti A., Augustin L.B., Modiano J.F. (2016). A phase I clinical study to evaluate safety of orally administered, genetically engineered Salmonella enterica serovar Typhimurium for canine osteosarcoma. Vet. Med. Sci..

[17] Heimann D.M., Rosenberg S.A. (2003). Continuous intravenous administration of live genetically modified salmonella typhimurium in patients with metastatic melanoma. J. Immunother..

[18] Nemunaitis J., Cunningham C., Senzer N., Kuhn J., Cramm J., Litz C., Cavagnolo R., Cahill A., Clairmont C., Sznol M. (2003). Pilot trial of genetically modified, attenuated Salmonella expressing the E. coli cytosine deaminase gene in refractory cancer patients. Cancer Gene Ther..

[19] Yu B., Yang M., Shi L., Yao Y., Jiang Q., Li X., Tang L.H., Zheng B.J., Yuen K.Y., Smith D.K. (2012). Explicit hypoxia targeting with tumor suppression by creating an "obligate" anaerobic Salmonella Typhimurium strain. Sci. Rep..

[20] Wei M.Q., Ellem K.A., Dunn P., West M.J., Bai C.X., Vogelstein B. (2007). Facultative or obligate anaerobic bacteria have the potential for multimodality therapy of solid tumours. Eur. J. Cancer.

[21] Martinez-Outschoorn U.E., Peiris-Pagés M., Pestell R.G., Sotgia F., Lisanti M.P. (2017). Cancer metabolism: A therapeutic perspective. Nat. Rev. Clin. Oncol..

[22] Sedighi M., Zahedi Bialvaei A., Hamblin M.R., Ohadi E., Asadi A., Halajzadeh M., Lohrasbi V., Mohammadzadeh N., Amiriani T., Krutova M. (2019). Therapeutic bacteria to combat cancer; current advances, challenges, and opportunities. Cancer Med..

[23] Jain R.K., Forbes N.S. (2001). Can engineered bacteria help control cancer?. Proc. Natl. Acad. Sci. USA.

[24] Carlisle R., Coussios C.C. (2013). Mechanical approaches to oncological drug delivery. Ther. Deliv..

[25] Brown J.M., Giaccia A.J. (1998). The unique physiology of solid tumors: Opportunities (and problems) for cancer therapy. Cancer Res..

[26] Cheong I., Zhou S. (2009). Tumor-Specific Liposomal Drug Release Mediated by Liposomase. Methods Enzymol..

[27] Nallar S.C., Xu D.Q., Kalvakolanu D.V. (2017). Bacteria and genetically modified bacteria as cancer therapeutics: Current advances and challenges. Cytokine.

[28] Yazawa K., Fujimori M., Amano J., Kano Y., Taniguchi S. (2000). Bifidobacterium longum as a delivery system for cancer gene therapy: Selective localization and growth in hypoxic tumors. Cancer Gene Ther..

[29] Chakrabarty A.M. (2003). Microorganisms and cancer: Quest for a therapy. J. Bacteriol..

[30] Song S., Vuai M.S., Zhong M. (2018). The role of bacteria in cancer therapy – enemies in the past, but allies at present. Infect. Agents Cancer.

[31] Park S.J., Park S.-H., Cho S., Kim D.-M., Lee Y., Ko S.Y., Hong Y., Choy H.E., Min J.-J., Park J.-O. (2013). New paradigm for tumor theranostic methodology using bacteria-based microrobot. Sci. Rep..

[32] Nguyen V.D., Han J.-W., Choi Y.J., Cho S., Zheng S., Ko S.Y., Park J.-O., Park S. (2016). Active tumor-therapeutic liposomal bacteriobot combining a drug (paclitaxel)-encapsulated liposome with targeting bacteria (Salmonella Typhimurium). Sens. Actuators B Chem..

[33] Park D., Park S.J., Cho S., Lee Y., Lee Y.K., Min J.-J., Park B.J., Ko S.Y., Park J.-O., Park S. (2014). Motility analysis of bacteria-based microrobot (bacteriobot) using chemical gradient microchamber. Biotechnol. Bioeng..

[34] Casadevall A., Pirofski L.-a. (2009). Virulence factors and their mechanisms of action: The view from a damage–response framework. J. Water Health.

[35] Cross A.S. (2008). What is a virulence factor?. Crit. Care.

[36] Lee C.H., Lin S.T., Liu J.J., Chang W.W., Hsieh J.L., Wang W.K. (2014). Salmonella induce autophagy in melanoma by the downregulation of AKT/mTOR pathway. Gene Ther..

[37] Frahm M., Felgner S., Kocijancic D., Rohde M., Hensel M., Curtiss R., Erhardt M., Weiss S. (2015). Efficiency of conditionally attenuated Salmonella enterica serovar Typhimurium in bacterium-mediated tumor therapy. mBio.

[38] Na H.S., Kim H.J., Lee H.C., Hong Y., Rhee J.H., Choy H.E. (2006). Immune response induced by Salmonella typhimurium defective in ppGpp synthesis. Vaccine.

[39] Glomski I.J., Gedde M.M., Tsang A.W., Swanson J.A., Portnoy D.A. (2002). The Listeria monocytogenes hemolysin has an acidic pH optimum to compartmentalize activity and prevent damage to infected host cells. J. Cell Biol..

[40] Glomski I.J., Decatur A.L., Portnoy D.A. (2003). Listeria monocytogenes mutants that fail to compartmentalize listerolysin O activity are cytotoxic, avirulent, and unable to evade host extracellular defenses. Infect. Immun..

[41] Camilli A., Tilney L.G., Portnoy D.A. (1993). Dual roles of plcA in Listeria monocytogenes pathogenesis. Mol. Microbiol..

[42] Decatur A.L., Portnoy D.A. (2000). A PEST-like sequence in listeriolysin O essential for Listeria monocytogenes pathogenicity. Science.

[43] Bakardjiev A.I., Stacy B.A., Fisher S.J., Portnoy D.A. (2004). Listeriosis in the pregnant guinea pig: A model of vertical transmission. Infect. Immun..

[44] Brockstedt D.G., Giedlin M.A., Leong M.L., Bahjat K.S., Gao Y., Luckett W., Liu W., Cook D.N., Portnoy D.A., Dubensky T.W. (2004). Listeria-based cancer vaccines that segregate immunogenicity from toxicity. Proc. Natl. Acad. Sci. USA.

[45] Chagnon A., Hudon C., McSween G., Vinet G., Fredette V. (1972). Cytotoxicity and reduction of animal cell growth by Clostridium M-55 spores and their extracts. Cancer.

[46] Cheong I., Huang X., Bettegowda C., Diaz L.A., Kinzler K.W., Zhou S., Vogelstein B. (2006). A bacterial protein enhances the release and efficacy of liposomal cancer drugs. Science.

[47] Felgner S., Kocijancic D., Frahm M., Weiss S. (2016). Bacteria in Cancer Therapy: Renaissance of an Old Concept. Int. J. Microbiol..

[48] Fronzes R., Christie P.J., Waksman G. (2009). The structural biology of type IV secretion systems. Nat. Rev. Microbiol..

[49] Singer H.M., Erhardt M., Steiner A.M., Zhang M.M., Yoshikami D., Bulaj G., Olivera B.M., Hughes K.T. (2012). Selective purification of recombinant neuroactive peptides using the flagellar type III secretion system. mBio.

[50] Farley M.M., Hu B., Margolin W., Liu J. (2016). Minicells, Back in Fashion. J. Bacteriol..

[51] Fensterle J., Bergmann B., Yone C.L., Hotz C., Meyer S.R., Spreng S., Goebel W., Rapp U.R., Gentschev I. (2008). Cancer immunotherapy based on recombinant Salmonella enterica serovar Typhimurium aroA strains secreting prostate-specific antigen and cholera toxin subunit B. Cancer Gene Ther..

[52] Nishikawa H., Sato E., Briones G., Chen L.M., Matsuo M., Nagata Y., Ritter G., Jäger E., Nomura H., Kondo S. (2006). In vivo antigen delivery by a Salmonella typhimurium type III secretion system for therapeutic cancer vaccines. J Clin. Investig..

[53] Panthel K., Meinel K.M., Sevil Domènech V.E., Geginat G., Linkemann K., Busch D.H., Rüssmann H. (2006). Prophylactic anti-tumor immunity against a murine fibrosarcoma triggered by the Salmonella type III secretion system. Microbes Infect..

[54] Roider E., Jellbauer S., Köhn B., Berchtold C., Partilla M., Busch D.H., Rüssmann H., Panthel K. (2011). Invasion and destruction of a murine fibrosarcoma by Salmonella-induced effector CD8 T cells as a therapeutic intervention against cancer. Cancer Immunol. Immunother..

[55] Epaulard O., Toussaint B., Quenee L., Derouazi M., Bosco N., Villiers C., Le Berre R., Guery B., Filopon D., Crombez L. (2006). Anti-tumor immunotherapy via antigen delivery from a live attenuated genetically engineered Pseudomonas aeruginosa type III secretion system-based vector. Mol. Ther..

[56] Paton A.W., Morona R., Paton J.C. (2012). Bioengineered microbes in disease therapy. Trends Mol. Med..

[57] Grillot-Courvalin C., Goussard S., Courvalin P. (2002). Wild-type intracellular bacteria deliver DNA into mammalian cells. Cell Microbiol..

[58] Akoachere J.F., Tanih N.F., Ndip L.M., Ndip R.N. (2009). Phenotypic characterization of Salmonella typhimurium isolates from food-animals and abattoir drains in Buea, Cameroon. J. Health Popul. Nutr..

[59] Mi Z., Feng Z.C., Li C., Yang X., Ma M.T., Rong P.F. (2019). Salmonella-Mediated Cancer Therapy: An Innovative Therapeutic Strategy. J. Cancer.

[60] Semenov A.V., van Overbeek L., Termorshuizen A.J., van Bruggen A.H. (2011). Influence of aerobic and anaerobic conditions on survival of Escherichia coli O157:H7 and Salmonella enterica serovar Typhimurium in Luria-Bertani broth, farm-yard manure and slurry. J. Environ. Manag..

[61] Loeffler M., Le’Negrate G., Krajewska M., Reed J.C. (2008). Inhibition of tumor growth using salmonella expressing Fas ligand. J. Natl. Cancer Inst..

[62] Ganai S., Arenas R.B., Forbes N.S. (2009). Tumour-targeted delivery of TRAIL using Salmonella typhimurium enhances breast cancer survival in mice. Br. J. Cancer.

[63] Yoon W.S., Chae Y.S., Hong J., Park Y.K. (2011). Antitumor therapeutic effects of a genetically engineered Salmonella typhimurium harboring TNF-α in mice. Appl. Microbiol. Biotechnol..

[64] Li Z., Yin P.H., Yang S.S., Li Q.Y., Chang T., Fang L., Shi L.X., Fang G.E. (2013). Recombinant attenuated Salmonella typhimurium carrying a plasmid co-expressing ENDO-VEGI151 and survivin siRNA inhibits the growth of breast cancer in vivo. Mol. Med. Rep..

[65] Lim D., Kim K.S., Kim H., Ko K.C., Song J.J., Choi J.H., Shin M., Min J.J., Jeong J.H., Choy H.E. (2017). Anti-tumor activity of an immunotoxin (TGFα-PE38) delivered by attenuated Salmonella typhimurium. Oncotarget.

[66] Mansour M., Ismail S., Abou-Aisha K. (2020). Bacterial delivery of the anti-tumor azurin-like protein Laz to glioblastoma cells. AMB Express.

[67] Gao S., Jung J.H., Lin S.M., Jang A.Y., Zhi Y., Bum Ahn K., Ji H.J., Hyang Lim J., Guo H., Choy H.E. (2020). Development of Oxytolerant Salmonella typhimurium Using Radiation Mutation Technology (RMT) for Cancer Therapy. Sci. Rep..

[68] Jiang S.N., Phan T.X., Nam T.K., Nguyen V.H., Kim H.S., Bom H.S., Choy H.E., Hong Y., Min J.J. (2010). Inhibition of tumor growth and metastasis by a combination of Escherichia coli-mediated cytolytic therapy and radiotherapy. Mol. Ther..

[69] Chiang C.-J., Hong Y.-H. (2021). In situ delivery of biobutyrate by probiotic Escherichia coli for cancer therapy. Sci. Rep..

[70] Zhang Y., Ji W., He L., Chen Y., Ding X., Sun Y., Hu S., Yang H., Huang W., Zhang Y. (2018). E. coli Nissle 1917-Derived Minicells for Targeted Delivery of Chemotherapeutic Drug to Hypoxic Regions for Cancer Therapy. Theranostics.

[71] Chowdhury S., Castro S., Coker C., Hinchliffe T.E., Arpaia N., Danino T. (2019). Programmable bacteria induce durable tumor regression and systemic antitumor immunity. Nat. Med..

[72] Radoshevich L., Cossart P. (2018). Listeria monocytogenes: Towards a complete picture of its physiology and pathogenesis. Nat. Rev. Microbiol..

[73] Wood L.M., Paterson Y. (2014). Attenuated Listeria monocytogenes: A powerful and versatile vector for the future of tumor immunotherapy. Front. Cell Infect. Microbiol..

[74] Hense M., Domann E., Krusch S., Wachholz P., Dittmar K.E., Rohde M., Wehland J., Chakraborty T., Weiss S. (2001). Eukaryotic expression plasmid transfer from the intracellular bacterium Listeria monocytogenes to host cells. Cell Microbiol..

[75] Akin D., Sturgis J., Ragheb K., Sherman D., Burkholder K., Robinson J.P., Bhunia A.K., Mohammed S., Bashir R. (2007). Bacteria-mediated delivery of nanoparticles and cargo into cells. Nat. Nanotechnol..

[76] van Pijkeren J.P., Morrissey D., Monk I.R., Cronin M., Rajendran S., O’Sullivan G.C., Gahan C.G., Tangney M. (2010). A novel Listeria monocytogenes-based DNA delivery system for cancer gene therapy. Hum. Gene Ther..

[77] Kim S.H., Castro F., Gonzalez D., Maciag P.C., Paterson Y., Gravekamp C. (2008). Mage-b vaccine delivered by recombinant Listeria monocytogenes is highly effective against breast cancer metastases. Br. J. Cancer.

[78] Kim Y.J., Perumalsamy H., Markus J., Balusamy S.R., Wang C., Ho Kang S., Lee S., Park S.Y., Kim S., Castro-Aceituno V. (2019). Development of Lactobacillus kimchicus DCY51(T)-mediated gold nanoparticles for delivery of ginsenoside compound K: In vitro photothermal effects and apoptosis detection in cancer cells. Artif. Cells Nanomed. Biotechnol..

[79] Kullberg M., McCarthy R., Anchordoquy T.J. (2014). Gene delivery to Her-2+ breast cancer cells using a two-component delivery system to achieve specificity. Nanomedicine.

[80] Gilert A., Baruch L., Bronshtein T., Machluf M. (2016). PLGA-Listeriolysin O microspheres: Opening the gate for cytosolic delivery of cancer antigens. Biomed. Microdevices.

[81] Mann K., Kullberg M. (2016). Trastuzumab-targeted gene delivery to Her2-overexpressing breast cancer cells. Cancer Gene Ther..

[82] Al-Ani A.W., Zhang L., Ferreira L., Turyanska L., Bradshaw T.D., Thomas N.R. (2019). Listeria innocua Dps as a nanoplatform for bioluminescence based photodynamic therapy utilizing Gaussia princeps luciferase and zinc protoporphyrin IX. Nanomedicine.

[83] Barbé S., Van Mellaert L., Anné J. (2006). The use of clostridial spores for cancer treatment. J. Appl. Microbiol..

[84] Van Mellaert L., Barbé S., Anné J. (2006). Clostridium spores as anti-tumour agents. Trends Microbiol..

[85] Mengesha A., Dubois L., Chiu R.K., Paesmans K., Wouters B.G., Lambin P., Theys J. (2007). Potential and limitations of bacterial-mediated cancer therapy. Front. Biosci..

[86] St Jean A.T., Zhang M., Forbes N.S. (2008). Bacterial therapies: Completing the cancer treatment toolbox. Curr. Opin. Biotechnol..

[87] Wei M.Q., Mengesha A., Good D., Anné J. (2008). Bacterial targeted tumour therapy-dawn of a new era. Cancer Lett..

[88] Zu C., Wang J. (2014). Tumor-colonizing bacteria: A potential tumor targeting therapy. Crit. Rev. Microbiol..

[89] Connell H.C. (1935). The Study and Treatment of Cancer by Proteolytic Enzymes: Preliminary Report. Can. Med. Assoc. J..

[90] Parker R.C., Plummer H.C., Siebenmann C.O., Chapman M.G. (1947). Effect of histolyticus infection and toxin on transplantable mouse tumors. Proc. Soc. Exp. Biol. Med..

[91] Fox M.E., Lemmon M.J., Mauchline M.L., Davis T.O., Giaccia A.J., Minton N.P., Brown J.M. (1996). Anaerobic bacteria as a delivery system for cancer gene therapy: In vitro activation of 5-fluorocytosine by genetically engineered clostridia. Gene Ther..

[92] Theys J., Nuyts S., Landuyt W., Van Mellaert L., Dillen C., Böhringer M., Dürre P., Lambin P., Anné J. (1999). Stable Escherichia coli-Clostridium acetobutylicum shuttle vector for secretion of murine tumor necrosis factor alpha. Appl. Environ. Microbiol..

[93] Barbé S., Van Mellaert L., Theys J., Geukens N., Lammertyn E., Lambin P., Anné J. (2005). Secretory production of biologically active rat interleukin-2 by Clostridium acetobutylicum DSM792 as a tool for anti-tumor treatment. FEMS Microbiol. Lett..

[94] Casares N., Pequignot M.O., Tesniere A., Ghiringhelli F., Roux S., Chaput N., Schmitt E., Hamai A., Hervas-Stubbs S., Obeid M. (2005). Caspase-dependent immunogenicity of doxorubicin-induced tumor cell death. J. Exp. Med..

[95] Michaud M., Martins I., Sukkurwala A.Q., Adjemian S., Ma Y., Pellegatti P., Shen S., Kepp O., Scoazec M., Mignot G. (2011). Autophagy-dependent anticancer immune responses induced by chemotherapeutic agents in mice. Science.

[96] Obeid M., Tesniere A., Ghiringhelli F., Fimia G.M., Apetoh L., Perfettini J.L., Castedo M., Mignot G., Panaretakis T., Casares N. (2007). Calreticulin exposure dictates the immunogenicity of cancer cell death. Nat. Med..

[97] Huang T., Li S., Li G., Tian Y., Wang H., Shi L., Perez-Cordon G., Mao L., Wang X., Wang J. (2014). Utility of Clostridium difficile toxin B for inducing anti-tumor immunity. PLoS ONE.

[98] Groot A.J., Verheesen P., Westerlaken E.J., Gort E.H., van der Groep P., Bovenschen N., van der Wall E., van Diest P.J., Shvarts A. (2006). Identification by phage display of single-domain antibody fragments specific for the ODD domain in hypoxia-inducible factor 1alpha. Lab. Investig..

[99] Collier R.J. (1975). Diphtheria toxin: Mode of action and structure. Bacteriol. Rev..

[100] Shafiee F., Aucoin M.G., Jahanian-Najafabadi A. (2019). Targeted Diphtheria Toxin-Based Therapy: A Review Article. Front. Microbiol..

[101] Shapira A., Benhar I. (2010). Toxin-based therapeutic approaches. Toxins.

[102] Zheng Q., Wang Z., Zhang H., Huang Q., Madsen J.C., Sachs D.H., Huang C.A., Wang Z. (2017). Diphtheria toxin-based anti-human CD19 immunotoxin for targeting human CD19(+) tumors. Mol. Oncol..

[103] Li Y.M., Hall W.A. (2010). Targeted toxins in brain tumor therapy. Toxins.

[104] Elsayad K., Kriz J., Moustakis C., Scobioala S., Reinartz G., Haverkamp U., Willich N., Weishaupt C., Stadler R., Sunderkötter C. (2015). Total Skin Electron Beam for Primary Cutaneous T-cell Lymphoma. Int. J. Radiat. Oncol.Biol.Phys..

[105] Zahaf N.I., Schmidt G. (2017). Bacterial Toxins for Cancer Therapy. Toxins.

[106] Kiyokawa T., Shirono K., Hattori T., Nishimura H., Yamaguchi K., Nichols J.C., Strom T.B., Murphy J.R., Takatsuki K. (1989). Cytotoxicity of interleukin 2-toxin toward lymphocytes from patients with adult T-cell leukemia. Cancer Res..

[107] Zhang Y., Schulte W., Pink D., Phipps K., Zijlstra A., Lewis J.D., Waisman D.M. (2010). Sensitivity of cancer cells to truncated diphtheria toxin. PLoS ONE.

[108] Leshem Y., Pastan I. (2019). Pseudomonas Exotoxin Immunotoxins and Anti-Tumor Immunity: From Observations at the Patient’s Bedside to Evaluation in Preclinical Models. Toxins.

[109] Michalska M., Wolf P. (2015). Pseudomonas Exotoxin A: Optimized by evolution for effective killing. Front. Microbiol..

[110] Wolf P., Elsässer-Beile U. (2009). Pseudomonas exotoxin A: From virulence factor to anti-cancer agent. Int. J. Med. Microbiol..

[111] Iglewski B.H., Liu P.V., Kabat D. (1977). Mechanism of action of Pseudomonas aeruginosa exotoxin Aiadenosine diphosphate-ribosylation of mammalian elongation factor 2 in vitro and in vivo. Infect. Immun..

[112] Kreitman R.J., Hassan R., Fitzgerald D.J., Pastan I. (2009). Phase I trial of continuous infusion anti-mesothelin recombinant immunotoxin SS1P. Clin. Cancer Res..

[113] Cheng X., Wang B., Jin Z., Ma D., Yang W., Zhao R., Jing X., Shen B., Peng C., Qiu W. (2016). Pseudomonas aeruginosa-mannose-sensitive hemagglutinin inhibits pancreatic cancer cell proliferation and induces apoptosis via the EGFR pathway and caspase signaling. Oncotarget.

[114] Li T., Dong Z.R., Guo Z.Y., Wang C.H., Zhi X.T., Zhou J.W., Li D.K., Chen Z.T., Chen Z.Q., Hu S.Y. (2015). Mannose-mediated inhibitory effects of PA-MSHA on invasion and metastasis of hepatocellular carcinoma via EGFR/Akt/IκBβ/NF-κB pathway. Liver Int..

[115] van Geel-Schutten G.H., Flesch F., ten Brink B., Smith M.R., Dijkhuizen L. (1998). Screening and characterization of Lactobacillus strains producing large amounts of exopolysaccharides. Appl. Microbiol. Biotechnol..

[116] Dethlefsen L., Huse S., Sogin M.L., Relman D.A. (2008). The pervasive effects of an antibiotic on the human gut microbiota, as revealed by deep 16S rRNA sequencing. PLoS Biol..

[117] Tamang J.P., Batt C.A., Tortorello M.L. (2014). BIOCHEMICAL AND MODERN IDENTIFICATION TECHNIQUES | Microfloras of Fermented Foods. Encyclopedia of Food Microbiology.

[118] Cano-Garrido O., Seras-Franzoso J., Garcia-Fruitós E. (2015). Lactic acid bacteria: Reviewing the potential of a promising delivery live vector for biomedical purposes. Microb Cell Fact..

[119] Oh N.S., Joung J.Y., Lee J.Y., Kim Y. (2018). Probiotic and anti-inflammatory potential of Lactobacillus rhamnosus 4B15 and Lactobacillus gasseri 4M13 isolated from infant feces. PLoS ONE.

[120] Hibberd A.A., Lyra A., Ouwehand A.C., Rolny P., Lindegren H., Cedgård L., Wettergren Y. (2017). Intestinal microbiota is altered in patients with colon cancer and modified by probiotic intervention. BMJ Open Gastroenterol..

[121] Tannock I.F., Rotin D. (1989). Acid pH in tumors and its potential for therapeutic exploitation. Cancer Res..

[122] Chang W.H., Liu J.J., Chen C.H., Huang T.S., Lu F.J. (2002). Growth inhibition and induction of apoptosis in MCF-7 breast cancer cells by fermented soy milk. Nutr. Cancer.

[123] Ohta T., Nakatsugi S., Watanabe K., Kawamori T., Ishikawa F., Morotomi M., Sugie S., Toda T., Sugimura T., Wakabayashi K. (2000). Inhibitory effects of Bifidobacterium-fermented soy milk on 2-amino-1-methyl-6-phenylimidazo[4,5-b]pyridine-induced rat mammary carcinogenesis, with a partial contribution of its component isoflavones. Carcinogenesis.

[124] Takagi A., Kano M., Kaga C. (2015). Possibility of breast cancer prevention: Use of soy isoflavones and fermented soy beverage produced using probiotics. Int. J. Mol. Sci..

[125] Dróżdż M., Makuch S., Cieniuch G., Woźniak M., Ziółkowski P. (2020). Obligate and facultative anaerobic bacteria in targeted cancer therapy: Current strategies and clinical applications. Life Sci..

[126] Park J., Kwon M., Lee J., Park S., Seo J., Roh S. (2020). Anti-Cancer Effects of Lactobacillus plantarum L-14 Cell-Free Extract on Human Malignant Melanoma A375 Cells. Molecules.

[127] Shida K., Nomoto K. (2013). Probiotics as efficient immunopotentiators: Translational role in cancer prevention. Indian J. Med. Res..

[128] Kamarajan P., Hayami T., Matte B., Liu Y., Danciu T., Ramamoorthy A., Worden F., Kapila S., Kapila Y. (2015). Nisin ZP, a Bacteriocin and Food Preservative, Inhibits Head and Neck Cancer Tumorigenesis and Prolongs Survival. PLoS ONE.

[129] Luo M., Hu M., Feng X., XiaoLi W., Dong D., Wang W. (2020). Preventive effect of Lactobacillus reuteri on melanoma. Biomed. Pharmacother..

[130] Yazdi M.H., Mahdavi M., Setayesh N., Esfandyar M., Shahverdi A.R. (2013). Selenium nanoparticle-enriched Lactobacillus brevis causes more efficient immune responses in vivo and reduces the liver metastasis in metastatic form of mouse breast cancer. Daru.

[131] Raj R., Das S. (2017). Development and application of anticancer fluorescent CdS nanoparticles enriched Lactobacillus bacteria as therapeutic microbots for human breast carcinoma. Appl. Microbiol. Biotechnol..

[132] Steidler L., Neirynck S., Huyghebaert N., Snoeck V., Vermeire A., Goddeeris B., Cox E., Remon J.P., Remaut E. (2003). Biological containment of genetically modified Lactococcus lactis for intestinal delivery of human interleukin 10. Nat. Biotechnol..

[133] Bahey-El-Din M., Gahan C.G., Griffin B.T. (2010). Lactococcus lactis as a cell factory for delivery of therapeutic proteins. Curr. Gene Ther..

[134] O’Callaghan A., van Sinderen D. (2016). Bifidobacteria and Their Role as Members of the Human Gut Microbiota. Front. Microbiol..

[135] Ngo N., Choucair K., Creeden J.F., Qaqish H., Bhavsar K., Murphy C., Lian K., Albrethsen M.T., Stanbery L., Phinney R.C. (2019). Bifidobacterium spp: The promising Trojan Horse in the era of precision oncology. Future Oncol..

[136] Wang L., Vuletic I., Deng D., Crielaard W., Xie Z., Zhou K., Zhang J., Sun H., Ren Q., Guo C. (2017). Bifidobacterium breve as a delivery vector of IL-24 gene therapy for head and neck squamous cell carcinoma in vivo. Gene Therapy.

[137] Wei C., Xun A.Y., Wei X.X., Yao J., Wang J.Y., Shi R.Y., Yang G.H., Li Y.X., Xu Z.L., Lai M.G. (2016). Bifidobacteria Expressing Tumstatin Protein for Antitumor Therapy in Tumor-Bearing Mice. Technol. Cancer Res. Treat..

[138] Li X., Fu G.-F., Fan Y.-R., Liu W.-H., Liu X.-J., Wang J.-J., Xu G.-X. (2003). Bifidobacterium adolescentis as a delivery system of endostatin for cancer gene therapy: Selective inhibitor of angiogenesis and hypoxic tumor growth. Cancer Gene Therapy.

[139] Fujimori M. (2006). Genetically engineered bifidobacterium as a drug delivery system for systemic therapy of metastatic breast cancer patients. Breast Cancer.

[140] Cronin M., Morrissey D., Rajendran S., El Mashad S.M., van Sinderen D., O’Sullivan G.C., Tangney M. (2010). Orally administered bifidobacteria as vehicles for delivery of agents to systemic tumors. Mol. Ther..

[141] Kikuchi T., Shimizu H., Akiyama Y., Taniguchi S. (2017). In situ delivery and production system of trastuzumab scFv with Bifidobacterium. Biochem. Biophys. Res. Commun..

[142] Bazylinski D.A., Williams T.J., Lefèvre C.T., Berg R.J., Zhang C.L., Bowser S.S., Dean A.J., Beveridge T.J. (2013). Magnetococcus marinus gen. nov., sp. nov., a marine, magnetotactic bacterium that represents a novel lineage (Magnetococcaceae fam. nov., Magnetococcales ord. nov.) at the base of the Alphaproteobacteria. Int. J. Syst. Evol. Microbiol..

[143] Afkhami F., Taherkhani S., Mohammadi M., Martel S. (2011). Encapsulation of magnetotactic bacteria for targeted and controlled delivery of anticancer agents for tumor therapy. Annu. Int. Conf. IEEE Eng. Med. Biol. Soc..

[144] Felfoul O., Mohammadi M., Taherkhani S., de Lanauze D., Zhong Xu Y., Loghin D., Essa S., Jancik S., Houle D., Lafleur M. (2016). Magneto-aerotactic bacteria deliver drug-containing nanoliposomes to tumour hypoxic regions. Nat. Nanotechnol..

[145] Zhao M., Yang M., Li X.M., Jiang P., Baranov E., Li S., Xu M., Penman S., Hoffman R.M. (2005). Tumor-targeting bacterial therapy with amino acid auxotrophs of GFP-expressing Salmonella typhimurium. Proc. Natl. Acad. Sci. USA.

[146] Clairmont C., Lee K.C., Pike J., Ittensohn M., Low K.B., Pawelek J., Bermudes D., Brecher S.M., Margitich D., Turnier J. (2000). Biodistribution and genetic stability of the novel antitumor agent VNP20009, a genetically modified strain of Salmonella typhimurium. J. Infect. Dis..

[147] Pawelek J.M., Low K.B., Bermudes D. (1997). Tumor-targeted Salmonella as a novel anticancer vector. Cancer Res..

[148] Zheng J.H., Min J.J. (2016). Targeted Cancer Therapy Using Engineered Salmonella typhimurium. Chonnam Med. J..

[149] Nguyen V.H., Kim H.S., Ha J.M., Hong Y., Choy H.E., Min J.J. (2010). Genetically engineered Salmonella typhimurium as an imageable therapeutic probe for cancer. Cancer Res..

[150] Zheng J.H., Nguyen V.H., Jiang S.N., Park S.H., Tan W., Hong S.H., Shin M.G., Chung I.J., Hong Y., Bom H.S. (2017). Two-step enhanced cancer immunotherapy with engineered Salmonella typhimurium secreting heterologous flagellin. Sci. Transl. Med..

[151] Meng J.Z., Dong Y.J., Huang H., Li S., Zhong Y., Liu S.L., Wang Y.D. (2010). Oral vaccination with attenuated Salmonella enterica strains encoding T-cell epitopes from tumor antigen NY-ESO-1 induces specific cytotoxic T-lymphocyte responses. Clin. Vaccine Immunol..

[152] Ahmad S., Casey G., Cronin M., Rajendran S., Sweeney P., Tangney M., O’Sullivan G.C. (2011). Induction of effective antitumor response after mucosal bacterial vector mediated DNA vaccination with endogenous prostate cancer specific antigen. J. Urol..

[153] al-Ramadi B.K., Fernandez-Cabezudo M.J., El-Hasasna H., Al-Salam S., Bashir G., Chouaib S. (2009). Potent anti-tumor activity of systemically-administered IL2-expressing Salmonella correlates with decreased angiogenesis and enhanced tumor apoptosis. Clin. Immunol..

[154] Felgner S., Frahm M., Kocijancic D., Rohde M., Eckweiler D., Bielecka A., Bueno E., Cava F., Abraham W.R., Curtiss R. (2016). aroA-Deficient Salmonella enterica Serovar Typhimurium Is More Than a Metabolically Attenuated Mutant. mBio.

[155] Rüssmann H., Shams H., Poblete F., Fu Y., Galán J.E., Donis R.O. (1998). Delivery of epitopes by the Salmonella type III secretion system for vaccine development. Science.

[156] Xiong G., Husseiny M.I., Song L., Erdreich-Epstein A., Shackleford G.M., Seeger R.C., Jäckel D., Hensel M., Metelitsa L.S. (2010). Novel cancer vaccine based on genes of Salmonella pathogenicity island 2. Int. J. Cancer.

[157] Shi L., Yu B., Cai C.H., Huang J.D. (2016). Angiogenic inhibitors delivered by the type III secretion system of tumor-targeting Salmonella typhimurium safely shrink tumors in mice. AMB Express.

[158] Sorenson B.S., Banton K.L., Frykman N.L., Leonard A.S., Saltzman D.A. (2008). Attenuated Salmonella typhimurium with IL-2 gene reduces pulmonary metastases in murine osteosarcoma. Clin. Orthop. Relat. Res..

[159] Lewēn S., Zhou H., Hu H.D., Cheng T., Markowitz D., Reisfeld R.A., Xiang R., Luo Y. (2008). A Legumain-based minigene vaccine targets the tumor stroma and suppresses breast cancer growth and angiogenesis. Cancer Immunol. Immunother..

[160] Jellbauer S., Panthel K., Hetrodt J.H., Rüssmann H. (2012). CD8 T-cell induction against vascular endothelial growth factor receptor 2 by Salmonella for vaccination purposes against a murine melanoma. PLoS ONE.

[161] Liang K., Liu Q., Li P., Han Y., Bian X., Tang Y., Kong Q. (2018). Endostatin gene therapy delivered by attenuated Salmonella typhimurium in murine tumor models. Cancer Gene Ther..

[162] Mesa-Pereira B., Medina C., Camacho E.M., Flores A., Santero E. (2015). Improved cytotoxic effects of Salmonella-producing cytosine deaminase in tumour cells. Microb Biotechnol..

[163] Gunn G.R., Zubair A., Peters C., Pan Z.K., Wu T.C., Paterson Y. (2001). Two Listeria monocytogenes vaccine vectors that express different molecular forms of human papilloma virus-16 (HPV-16) E7 induce qualitatively different T cell immunity that correlates with their ability to induce regression of established tumors immortalized by HPV-16. J. Immunol..

[164] Shahabi V., Reyes-Reyes M., Wallecha A., Rivera S., Paterson Y., Maciag P. (2008). Development of a Listeria monocytogenes based vaccine against prostate cancer. Cancer Immunol. Immunother..

[165] Carvalho F., Sousa S., Cabanes D. (2014). How Listeria monocytogenes organizes its surface for virulence. Front. Cell Infect. Microbiol..

[166] Rafelski S.M., Theriot J.A. (2006). Mechanism of polarization of Listeria monocytogenes surface protein ActA. Mol. Microbiol..

[167] Sewell D.A., Shahabi V., Gunn G.R., Pan Z.K., Dominiecki M.E., Paterson Y. (2004). Recombinant Listeria vaccines containing PEST sequences are potent immune adjuvants for the tumor-associated antigen human papillomavirus-16 E7. Cancer Res..

[168] Olino K., Wada S., Edil B.H., Pan X., Meckel K., Weber W., Slansky J., Tamada K., Lauer P., Brockstedt D. (2012). Tumor-associated antigen expressing Listeria monocytogenes induces effective primary and memory T-cell responses against hepatic colorectal cancer metastases. Ann. Surg. Oncol..

[169] Johnson P.V., Blair B.M., Zeller S., Kotton C.N., Hohmann E.L. (2011). Attenuated Listeria monocytogenes vaccine vectors expressing influenza A nucleoprotein: Preclinical evaluation and oral inoculation of volunteers. Microbiol. Immunol..

[170] Morales A., Eidinger D., Bruce A.W. (1976). Intracavitary Bacillus Calmette-Guerin in the treatment of superficial bladder tumors. J. Urol..

[171] Biot C., Rentsch C.A., Gsponer J.R., Birkhäuser F.D., Jusforgues-Saklani H., Lemaître F., Auriau C., Bachmann A., Bousso P., Demangel C. (2012). Preexisting BCG-specific T cells improve intravesical immunotherapy for bladder cancer. Sci. Transl. Med..

[172] Roberts N.J., Zhang L., Janku F., Collins A., Bai R.Y., Staedtke V., Rusk A.W., Tung D., Miller M., Roix J. (2014). Intratumoral injection of Clostridium novyi-NT spores induces antitumor responses. Sci. Transl. Med..

[173] Theys J., Lambin P. (2015). Clostridium to treat cancer: Dream or reality?. Ann. Transl. Med..

[174] Dang L.H., Bettegowda C., Huso D.L., Kinzler K.W., Vogelstein B. (2001). Combination bacteriolytic therapy for the treatment of experimental tumors. Proc. Natl. Acad. Sci. USA.

[175] Park B.W., Zhuang J., Yasa O., Sitti M. (2017). Multifunctional Bacteria-Driven Microswimmers for Targeted Active Drug Delivery. ACS Nano.

[176] Ohta N., Fukase S., Watanabe T., Ito T., Aoyagi M. (2010). Effects and mechanism of OK-432 therapy in various neck cystic lesions. Acta Otolaryngol..

[177] Ohta N., Fukase S., Suzuki Y., Ishida A., Aoyagi M. (2010). Treatments of various otolaryngological cystic diseases by OK-4321: Its indications and limitations. Laryngoscope.

[178] Kono M., Satomi T., Abukawa H., Hasegawa O., Watanabe M., Chikazu D. (2017). Evaluation of OK-432 Injection Therapy as Possible Primary Treatment of Intraoral Ranula. J. Oral Maxillofac. Surg.

[179] Ogita S., Tsuto T., Nakamura K., Deguchi E., Tokiwa K., Iwai N. (1996). OK-432 therapy for lymphangioma in children: Why and how does it work?. J. Pediatr. Surg..

[180] Veena V.K., Popavath R.N., Kennedy K., Sakthivel N. (2015). In vitro antiproliferative, pro-apoptotic, antimetastatic and anti-inflammatory potential of 2,4-diacteylphloroglucinol (DAPG) by Pseudomonas aeruginosa strain FP10. Apoptosis.

[181] Riaz Rajoka M.S., Zhao H., Mehwish H.M., Li N., Lu Y., Lian Z., Shao D., Jin M., Li Q., Zhao L. (2019). Anti-tumor potential of cell free culture supernatant of Lactobacillus rhamnosus strains isolated from human breast milk. Food Res. Int..

[182] Liu C.F., Pan T.M. (2010). In Vitro Effects of Lactic Acid Bacteria on Cancer Cell Viability and Antioxidant Activity. J. Food Drug Anal..

[183] Asoudeh-Fard A., Barzegari A., Dehnad A., Bastani S., Golchin A., Omidi Y. (2017). Lactobacillus plantarum induces apoptosis in oral cancer KB cells through upregulation of PTEN and downregulation of MAPK signalling pathways. Bioimpacts.

[184] Abdolalipour E., Mahooti M., Salehzadeh A., Torabi A., Mohebbi S.R., Gorji A., Ghaemi A. (2020). Evaluation of the antitumor immune responses of probiotic Bifidobacterium bifidum in human papillomavirus-induced tumor model. Microb. Pathog..

[185] Wang Q., Wang K., Wu W., Lv L., Bian X., Yang L., Wang Q., Li Y., Ye J., Fang D. (2020). Administration of Bifidobacterium bifidum CGMCC 15068 modulates gut microbiota and metabolome in azoxymethane (AOM)/dextran sulphate sodium (DSS)-induced colitis-associated colon cancer (CAC) in mice. Appl. Microbiol. Biotechnol..

[186] Kitagawa K., Gonoi R., Tatsumi M., Kadowaki M., Katayama T., Hashii Y., Fujisawa M., Shirakawa T. (2019). Preclinical Development of a WT1 Oral Cancer Vaccine Using a Bacterial Vector to Treat Castration-Resistant Prostate Cancer. Mol. Cancer Ther..

[187] Coley W.B. (1891). Contribution to the Knowledge of Sarcoma. Ann. Surg.

[188] Heppner F., Möse J.R. (1978). The liquefaction (oncolysis) of malignant gliomas by a non pathogenic Clostridium. Acta Neurochir (Wien.).

[189] Basu P., Mehta A., Jain M., Gupta S., Nagarkar R.V., John S., Petit R. (2018). A Randomized Phase 2 Study of ADXS11-001 Listeria monocytogenes-Listeriolysin O Immunotherapy With or Without Cisplatin in Treatment of Advanced Cervical Cancer. Int. J. Gynecol Cancer.

[190] Le D.T., Wang-Gillam A., Picozzi V., Greten T.F., Crocenzi T., Springett G., Morse M., Zeh H., Cohen D., Fine R.L. (2015). Safety and survival with GVAX pancreas prime and Listeria Monocytogenes-expressing mesothelin (CRS-207) boost vaccines for metastatic pancreatic cancer. J. Clin. Oncol..

[191] Gniadek T.J., Augustin L., Schottel J., Leonard A., Saltzman D., Greeno E., Batist G. (2020). A Phase I, Dose Escalation, Single Dose Trial of Oral Attenuated Salmonella typhimurium Containing Human IL-2 in Patients With Metastatic Gastrointestinal Cancers. J. Immunother..

[192] Schmitz-Winnenthal F.H., Hohmann N., Schmidt T., Podola L., Friedrich T., Lubenau H., Springer M., Wieckowski S., Breiner K.M., Mikus G. (2018). A phase 1 trial extension to assess immunologic efficacy and safety of prime-boost vaccination with VXM01, an oral T cell vaccine against VEGFR2, in patients with advanced pancreatic cancer. Oncoimmunology.

[193] Griffin M.E., Espinosa J., Becker J.L., Luo J.D., Carroll T.S., Jha J.K., Fanger G.R., Hang H.C. (2021). Enterococcus peptidoglycan remodeling promotes checkpoint inhibitor cancer immunotherapy. Science.

[194] Duong M.T.-Q., Qin Y., You S.-H., Min J.-J. (2019). Bacteria-cancer interactions: Bacteria-based cancer therapy. Exp. Mol. Med..

[195] Miyake K., Murata T., Murakami T., Zhao M., Kiyuna T., Kawaguchi K., Igarashi K., Miyake M., Lwin T.M., Hozumi C. (2019). Tumor-targeting Salmonella typhimurium A1-R overcomes nab-paclitaxel resistance in a cervical cancer PDOX mouse model. Arch. Gynecol. Obstet..

